# Composite type-2 biomarker strategy versus a symptom–risk-based algorithm to adjust corticosteroid dose in patients with severe asthma: a multicentre, single-blind, parallel group, randomised controlled trial

**DOI:** 10.1016/S2213-2600(20)30397-0

**Published:** 2021-01

**Authors:** Liam G Heaney, John Busby, Catherine E Hanratty, Ratko Djukanovic, Ashley Woodcock, Samantha M Walker, Timothy C Hardman, Joseph R Arron, David F Choy, Peter Bradding, Christopher E Brightling, Rekha Chaudhuri, Douglas C Cowan, Adel H Mansur, Stephen J Fowler, Robert M Niven, Peter H Howarth, James L Lordan, Andrew Menzies-Gow, Tim W Harrison, Douglas S Robinson, Cecile T J Holweg, John G Matthews, Ian D Pavord, investigators for the MRC Refractory Asthma Stratification Programme, Ian M Adcock, Adnam Azim, Mary Bellamy, Catherine Borg, Michelle Bourne, Clare Connolly, Richard W Costello, Chris J Corrigan, Sarah Davies, Gareth Davies, Kian F Chung, Gabrielle Gainsborough, Traceyanne Grandison, Beverley Hargadon, Avril Horn, Val Hudson, David Jackson, Sebastian Johnston, Geraldine Jones, Paula McCourt, Maria Nunez, Dominic E Shaw, Katherine Smith, Joel Solis, Roisin Stone, Freda Yang

**Affiliations:** a Centre for Experimental Medicine, School of Medicine, Dentistry and Biomedical Sciences, Queen's University, Belfast, UK; b 23andMe, Sunnyvale, CA, USA; c Oxford Respiratory NIHR BRC, Nuffield Department of Medicine, University of Oxford, Oxford, UK; d Genentech, South San Francisco, CA, USA; e Department of Respiratory Sciences, Institute for Lung Health and Leicester NIHR Biomedical Research Centre, University of Leicester, Leicester, UK; f NHS Greater Glasgow and Clyde Health Board, Gartnavel Hospital, and University of Glasgow, Glasgow, UK; g NHS Greater Glasgow and Clyde, Stobhill Hospital, Glasgow, UK; h Division of Infection, Immunity and Respiratory Medicine, School of Biological Sciences, University of Manchester and Manchester Academic Health Science Centre and NIHR Manchester Biomedical Research Centre, Manchester University Hospitals NHS Foundation Trust, Manchester, UK; i Niche Science & Technology, Falstaff House, Richmond, UK; j Nottingham Respiratory NIHR Biomedical Research Centre, University of Nottingham, Nottingham, UK; k School of Clinical and Experimental Sciences, University of Southampton, NIHR Southampton Biomedical Research Centre, Southampton, UK; l The Newcastle upon Tyne NHS Foundation Trust, Newcastle upon Tyne, UK; m University of Birmingham and Heartlands Hospital, University Hospitals Birmingham NHS Foundation Trust, Birmingham, UK; n Royal Brompton & Harefield NHS Foundation Trust, London, UK; o University College Hospitals NHS Foundation Trust, London, UK; p Asthma UK & British Lung Foundation Partnership, London, UK

## Abstract

**Background:**

Asthma treatment guidelines recommend increasing corticosteroid dose to control symptoms and reduce exacerbations. This approach is potentially flawed because symptomatic asthma can occur without corticosteroid responsive type-2 (T2)-driven eosinophilic inflammation, and inappropriately high-dose corticosteroid treatment might have little therapeutic benefit with increased risk of side-effects. We compared a biomarker strategy to adjust corticosteroid dose using a composite score of T2 biomarkers (fractional exhaled nitric oxide [FENO], blood eosinophils, and serum periostin) with a standardised symptom–risk-based algorithm (control).

**Methods:**

We did a single-blind, parallel group, randomised controlled trial in adults (18–80 years of age) with severe asthma (at treatment steps 4 and 5 of the Global Initiative for Asthma) and FENO of less than 45 parts per billion at 12 specialist severe asthma centres across England, Scotland, and Northern Ireland. Patients were randomly assigned (4:1) to either the biomarker strategy group or the control group by an online electronic case-report form, in blocks of ten, stratified by asthma control and use of rescue systemic steroids in the previous year. Patients were masked to study group allocation throughout the entirety of the study. Patients attended clinic every 8 weeks, with treatment adjustment following automated treatment-group-specific algorithms: those in the biomarker strategy group received a default advisory to maintain treatment and those in the control group had their treatment adjusted according to the steps indicated by the trial algorithm. The primary outcome was the proportion of patients with corticosteroid dose reduction at week 48, in the intention-to-treat (ITT) population. Secondary outcomes were inhaled corticosteroid (ICS) dose at the end of the study; cumulative dose of ICS during the study; proportion of patients on maintenance oral corticosteroids (OCS) at study end; rate of protocol-defined severe exacerbations per patient year; time to first severe exacerbation; number of hospital admissions for asthma; changes in lung function, Asthma Control Questionnaire-7 score, Asthma Quality of Life Questionnaire score, and T2 biomarkers from baseline to week 48; and whether patients declined to progress to OCS. A secondary aim of our study was to establish the proportion of patients with severe asthma in whom T2 biomarkers remained low when corticosteroid therapy was decreased to a minimum ICS dose. This study is registered with ClinicalTrials.gov, NCT02717689 and has been completed.

**Findings:**

Patients were recruited from Jan 8, 2016, to July 12, 2018. Of 549 patients assessed, 301 patients were included in the ITT population and were randomly assigned to the biomarker strategy group (n=240) or to the control group (n=61). 28·4% of patients in the biomarker strategy group were on a lower corticosteroid dose at week 48 compared with 18·5% of patients in the control group (adjusted odds ratio [aOR] 1·71 [95% CI 0·80–3·63]; p=0·17). In the per-protocol (PP) population (n=121), a significantly greater proportion of patients were on a lower corticosteroid dose at week 48 in the biomarker strategy group (30·7% of patients) compared with the control group (5·0% of patients; aOR 11·48 [95% CI 1·35–97·83]; p=0·026). Patient choice to not follow treatment advice was the principle reason for loss to PP analysis. There was no difference in secondary outcomes between study groups and no loss of asthma control among patients in the biomarker strategy group who reduced their corticosteroid dose.

**Interpretation:**

Biomarker-based corticosteroid adjustment did not result in a greater proportion of patients reducing corticosteroid dose versus control. Understanding the reasons for patients not following treatment advice in both treatment strategies is an important area for future research. The prevalence of T2 biomarker-low severe asthma was low.

**Funding:**

This study was funded, in part, by the Medical Research Council UK.

**Research in context**

**Evidence before this study**

Treatment guidelines for asthma recommend a step-wise increase of corticosteroid treatment to control symptoms and reduce exacerbations. Evidence suggests minimal therapeutic benefit from corticosteroids in the absence of type 2 (T2)-driven eosinophilic inflammation, and in many symptomatic patients with asthma treated with corticosteroids, this type of inflammation is not present. Biomarker-based adjustment of corticosteroid treatment in severe asthma by means of sputum eosinophilia reduces exacerbations and allows safe down-titration of corticosteroid treatment, but is difficult to deliver in real-world settings. To our knowledge, no previous study has considered a biomarker-based strategy with the primary aim to reduce corticosteroid treatment in patients with severe asthma on high-dose treatment. Guidelines advocate corticosteroid dose reduction based on a combination of exacerbation risk and symptom control but evidence to support use of this strategy in severe asthma is scarce.

**Added value of this study**

We found no significant difference in the proportion of patients who had reduced corticosteroid dose at week 48 between those who were assigned to the T2 biomarker strategy compared with those assigned to standard care. A high proportion of patients chose not to follow study treatment advice but in those who did, a significant benefit was seen in patients in the biomarker-based strategy group. Importantly, patients who reduced treatment according to biomarker-directed therapy showed no evidence of clinical deterioration, despite being on lower corticosteroid doses.

**Implications of all the available evidence**

Factors driving patient choice not to follow advice to reduce corticosteroid treatment are an important area for future research. We suggest that before progression to high-dose corticosteroid treatment, predictive biomarkers of therapeutic response should be assessed to guide treatment decisions, because once a patient becomes established on high-dose corticosteroid treatment, biomarker-driven corticosteroid reduction can be difficult to achieve in symptomatic patients.

## Introduction

Treatment guidelines for asthma recommend a step-wise increase in inhaled corticosteroid (ICS) treatment to control symptoms and reduce exacerbations.^1^ However, as many as 50% of patients with symptomatic asthma might not have eosinophilic airways inflammation and respond poorly to corticosteroids.^2^, ^3^, ^4^ In this population, there is a clear potential for treatment to be increased to high intensity ICS and oral corticosteroids (OCS) without therapeutic benefit and with an increased potential for side-effects.^5^, ^6^, ^7^, ^8^, ^9^

Titrating corticosteroids with the goal of minimising sputum eosinophilia reduces severe exacerbations and allows safe down-titration of corticosteroid treatment in severe asthma.^10^, ^11^, ^12^ However, sputum-guided management is difficult to deliver in a real-world setting. When used as a single biomarker of type-2 (T2) cytokine inflammation, fractional exhaled nitric oxide (FENO)-guided management in mild and moderate asthma results in reduced exacerbations with minimal effect on lung function or asthma symptoms.^12^ However, whether a biomarker-based strategy can be used to primarily reduce corticosteroid exposure in patients with severe asthma on high-dose treatment is unclear. Asthma guidelines advocate corticosteroid reduction by assessment of exacerbation risk and symptom control but there is little evidence base to support this strategy in patients with severe asthma on high-dose corticosteroid treatment, particularly in patients receiving OCS.^1^ Given that high doses of corticosteroid cause considerable morbidity, new algorithms that can be delivered in the clinic for guiding corticosteroid treatment in patients with severe asthma are needed.

We have shown previously that a raised composite score of three T2-inflammation biomarkers (FENO, blood eosinophils, and serum periostin) is independently associated with increased risk of exacerbation in patients with severe asthma.^13^ This suggests that a low composite score could be used to identify a low-risk population in whom corticosteroid dose could be safely reduced.^13^ In this randomised, multicentre, parallel group trial in patients with severe asthma, we aimed to show whether such a scoring system could be used to guide corticosteroid treatment better than a standardised symptom-based or risk-based strategy, and would do so more effectively without increasing exacerbation rates, worsening asthma control, or precipitating a fall in lung function. An important secondary aim of our study was to establish the proportion of patients with severe asthma in whom T2 biomarkers remain low when corticosteroid therapy is decreased to a minimum ICS dose.

## Methods

### Study design and participants

This was a multicentre, single-blind (study participant), parallel group randomised controlled trial in patients with severe asthma. Details of the protocol have been published elsewhere.^14^ In brief, we compared a composite biomarker-based adjustment of corticosteroid therapy (using a composite index of blood eosinophil count, serum periostin, and FENO concentration; active intervention) with adjustments using an algorithm based on asthma symptoms, lung function, and recent exacerbation history (control). The Medical Research Council Refractory Asthma Stratification Programme research consortium included a patient input platform recruited to provide direction with regard to patient needs and understanding; the group was recruited through Asthma UK.

The study was done in patients with severe asthma (Global Initiative for Asthma steps 4 and 5 classification of asthma severity) attending one of 12 specialist severe asthma centres across the UK (appendix pp 2–3). Patients eligible for enrolment were aged 18–80 years, had met well characterised diagnostic criteria for severe asthma, and had an FENO of less than 45 parts per billion (ppb) to enrich for a T2 biomarker-low population with greater opportunity to reduce corticosteroid treatment (appendix pp 5–6). Full eligibility criteria are included in the trial protocol (appendix pp 5–6).

The protocol was reviewed and approved by the Office for Research Ethics Northern Ireland (NI0158) and obtained local National Health Service Research and Development approval for individual sites. All patients provided written informed consent for study participation.

### Randomisation and masking

Following a 2-week run-in period, patients were randomly assigned in a 4:1 ratio to one of the two treatment groups: biomarker-based adjustment strategy or control adjustment strategy. This randomisation ratio was employed to assist study recruitment to the biomarker-guided treatment strategy and to ensure maximal numbers of patients undergoing biomarker-based corticosteroid treatment adjustment (to establish the proportion of patients who remained T2 biomarker-low when corticosteroid doses were reduced). The online electronic case-report form (Dendrite Clinical Systems, Reading, UK) assigned patients following a random schedule to one of the treatment groups in blocks of ten, stratified by asthma control (Asthma Control Questionnaire [ACQ-7] ≥1·5) and use of rescue systemic steroids (≥2 courses in previous year). This was a single-blind study with patients masked to their allocated treatment group throughout the study.

### Procedures

Following randomisation, patients attended the clinic every 8 weeks for review of their asthma control and treatment. Patient ACQ-7 scores, post-bronchodilator FEV_1_, and FENO concentration data were entered into the electronic case-report form at all study visits; blood eosinophil count data were entered into the electronic case-report form within 24 h of the visit; and periostin values were entered automatically by a central laboratory within 3–5 days of sample collection (or when available). The electronic case-report form software processed individual patient data by means of the study algorithms (Table 1, Table 2) and generated a treatment advisory in both treatment groups (ie, recommendations for therapeutic adjustment as appropriate to decrease, maintain, or increase treatment; appendix, pp 6–8).

**Table 1:** Composite biomarker scoring system

	**0**	**1**	**2**
FENO, ppb	<15	15–30	>30
Blood eosinophil count, n/μL	<150	150–300	>300
Periostin, ng/mL	<45	45–55	>55

FENO, blood eosinophil count, and serum periostin were measured at each study visit with each biomarker assigned a score of 0, 1, or 2. The composite biomarker score was calculated automatically by the eCRF software using the rounded average of the sum of all three biomarker scores. Treatment algorithms were generated automatically by the eCRF software; a composite biomarker score of 0 advised treatment reduction, a score of 1 advised maintenance of current treatment, and a score of 2 advised treatment increase. ppb=parts per billion. FENO=fractional exhaled nitric oxide. eCRF= electronic case-report form.

**Table 2:** Symptom–risk-based treatment adjustment

	**Score**
ACQ-7 ≥1·5 and ≥1 change from baseline score or a severe exacerbation since last study visit (previous 8 weeks at baseline randomisation visit)	2
ACQ-7 is 1·0 to <1·5 or ACQ-7 ≥1·5 and <1 change from baseline score AND no severe exacerbation since last study visit (previous 8 weeks at baseline randomisation visit)	1
ACQ-7 <1·0 and no severe exacerbation since last study visit (previous 8 weeks at baseline randomisation visit)	0

ACQ-7 and recent exacerbation history were recorded at each study visit. To mirror usual clinical care, patients were not asked to withhold bronchodilator medication before study spirometry measurements. Treatment algorithms were generated automatically by the eCRF software; this was considered essential as prestudy observations in the UK Severe Asthma Registry had identified standard care and specifically corticosteroid treatment regimes differed substantially across clinical centres. A score of 0 advised treatment reduction, a score of 1 advised maintenance of current treatment and a score of 2 advised treatment increase. ACQ-7=Asthma Control Questionnaire-7. eCRF= electronic case-report form.

Patients in both groups received instructions at each clinic visit from dedicated study coordinators trained in the study procedures and working closely with study investigators; those in the biomarker strategy group received a default advisory to maintain treatment and those in the control group had their treatment adjusted according to the steps indicated by the trial algorithm. All participants were contacted once biomarker results were available, thereby ensuring patients remained masked to which treatment group they were in; patients in the control group received a default advisory to maintain their treatment, whereas treatment advice in the biomarker group was based on the result generated by the trial algorithm. A default advisory to maintain treatment was provided if, for practical reasons, any individual biomarker measure was not available. When patients reported an asthma exacerbation, it was managed according to the patient's self-management plan with no adjustment in background treatment. Planned therapy adjustments were deferred until the next scheduled study visit. Patients remained in the study irrespective of whether or not they followed their treatment advisories. Patients were seen by the clinical investigator in the clinic if there was any concern about persistent poorly controlled asthma or treatment adjustments.

On completion of the study at week 48, all patients underwent final assessment. In addition to standard sample collection, patients were asked to identify which treatment strategy they thought they had been assigned to.

### Outcomes

The primary outcome was the proportion of patients with a reduction in ICS or OCS dose from baseline to week 48. Secondary outcomes were the ICS dose at the end of the study, cumulative dose of ICS during the study, and proportion of patients on maintenance OCS at study end. Asthma outcomes were the rate of protocol-defined severe exacerbations per patient year, time to first severe exacerbation, number of hospital admissions for asthma, changes in lung function, ACQ-7 score, Asthma Quality of Life Questionnaire (AQLQ) score, and T2 biomarkers from baseline to week 48. We expected that some patients would be reluctant to progress to OCS irrespective of their biomarker or symptom score status, so this reluctance was a prespecified secondary outcome. To facilitate the analysis, detailed information was collected on whether treatment advisories were followed at each study visit with reasons for non-compliance recorded where possible. A severe asthma exacerbation was recorded as new or as increased asthma symptoms if it led to at least one of the following: a doubling of daily OCS dose (for patients on maintenance OCS); the prescription of a course of rescue OCS for 3 or more consecutive days; administration of intravenous or intramuscular corticosteroid for asthma; or hospital visit for asthma. Patients were asked to manage exacerbations according to their written personalised self-management plans that were provided as part of their routine clinical care. We did a post-hoc analysis to explore the effects of biomarker-based management in patients with uncontrolled asthma.

### Statistical analysis

It was assumed that a 20% difference between the study groups in the proportion of patients receiving a lower dose of corticosteroid treatment would be clinically meaningful. This assumption was supported by the findings of a subsequent Delphi exercise of international severe asthma experts.^15^ Assuming a study dropout rate of approximately 20% and a 10% reduction in the number of patients achieving a lower dose of corticosteroid in the control group, we estimated that 300 patients, randomly assigned in a 4:1 ratio, would provide the study with 80% power to show a 20% difference in corticosteroid reduction between the treatment groups.

Analysis was done according to a prespecified statistical analysis plan under the intention-to-treat (ITT) principle; two-tailed hypothesis tests were done at the 5% α-level with 95% CIs used throughout. For the primary outcome, reductions in corticosteroid doses between the start and end of the study were analysed by means of logistic regression models, adjusted for age, sex, smoking status, treatment centre, use of rescue steroids in the year before randomisation (categorised as <2 and ≥2 courses) and ACQ-7 score (categorised as <1·5 and ≥1·5) at baseline. Multiple imputation with chained equations was used, which assumed that data were missing at random with imputation models including treatment group, age, sex, smoking status, treatment centre, rescue steroids in the year before randomisation (categorised as <2 and ≥2 courses), ACQ-7 score (categorised as <1·5 and ≥1·5) at baseline, and change in corticosteroid dose between baseline and the first visit.^16^

All patients who attended at least one follow-up visit were included in the primary outcome analysis. Secondary outcomes were analysed by means of regression. Linear models were used for ACQ-7, AQLQ, FEV_1_, log (FENO), log [blood eosinophils], periostin, ICS dose, and OCS dose. Negative binomial models were used for protocol-defined exacerbation and hospitalisation counts. Logistic models were used for the probability of refusing to start OCS, and Cox proportional hazards models were used for the time to first protocol-defined exacerbation. All models were adjusted for age, sex, smoking status, treatment centre, rescue steroid use in the year before randomisation (categorised as <2 and ≥2 courses), and ACQ-7 score at baseline (categorised as <1·5 and ≥1·5). Data for outcomes measured at each study visit (ACQ-7, AQLQ, FEV_1_, FENO, blood eosinophil count, periostin, ICS dose, and OCS dose) were further adjusted for the baseline measurement of the outcome. Secondary analysis was only done on data from patients who completed the study without imputation of missing data.

The prespecified per-protocol (PP) analysis excluded data from patients who did not attend a study visit or who did not follow any study treatment advisory (except in instances where patients had low cortisol or if patients were already on the lowest ICS dose allowed according to the protocol). The PP analysis adjusted for the same set of variables as the primary analyses. Exploratory post-hoc analyses were done to understand the observed difference between the results seen in the ITT population versus the PP population: analysis restricted to patients who had uncontrolled asthma at study entry (defined as an ACQ-7 ≥1·5) and exacerbation rate in patients not following treatment advisories compared with those following treatment advisories. We assessed the effect of the imputation on our primary outcome by repeating our analysis under a complete-case framework. Additionally, we assessed the robustness of our findings to missing-not-at-random mechanisms by assuming a best-case scenario (assuming all withdrawals would have reduced their corticosteroid dose) and worst-case scenario (assuming all withdrawals would not have reduced their corticosteroid dose). All analyses were done with the STATA 16 software package (StataCorp, TX, USA). Conduct of the trial was monitored by an independent trial steering committee. This study is registered with ClinicalTrials.gov, NCT02717689.

### Role of the funding source

The funder of the study had no role in study design, data collection, data analysis, data interpretation, or writing of the report. All authors had full access to all the data in the study and had final responsibility for the decision to submit for publication.

## Results

**Figure 1:**
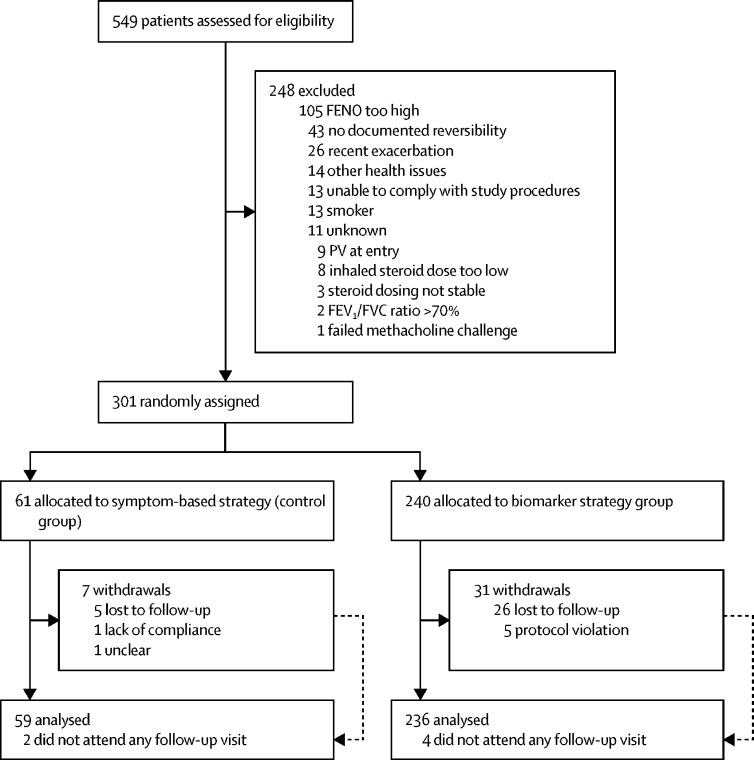
Trial profile FENO=fractional exhaled nitric oxide. PV=protocol violation. FVC=forced vital capacity.

Patients were recruited from Jan 8, 2016, to July 12, 2018 (figure 1). Of 549 patients assessed, 301 were randomly assigned to the biomarker strategy group (n=240) or to the control group (n=61). Baseline demographics, medical history, comorbidities, lung function, and corticosteroid treatment at baseline are shown in table 3.

**Table 3:** Demographics, medical history, lung function, biomarkers, corticosteroid treatment, and patient reported outcomes in the randomised population

		**Biomarker strategy group (n=240)**	**Control group (n=61)**
Age at inclusion, years		55·2 (13·4)	57·5 (11·9)
Sex			
	Female	151 (63%)	43 (70%)
	Male	89 (37%)	18 (30%)
Ethnicity*			
	Caucasian	221 (92%)	58 (95%)
	Non-Caucasian	19 (8%)	3 (5%)
Body-mass index, kg/m^2^		31·4 (7·2)	32·5 (7·2)
Smoking status			
	Never smoked	183 (76%)	41 (67%)
	Ex-smoker	57 (24%)	20 (33%)
Working status			
	Not working owing to asthma-related ill health	39 (16%)	13 (21%)
	Not working owing to other cause	76 (32%)	26 (43%)
	Working part-time owing to asthma-related ill health	12 (5%)	1 (2%)
	Working part-time owing to other cause	38 (16%)	1 (2%)
	Student	2 (1%)	0
	Full time	72 (30%)	20 (33%)
Atopic disease		167 (70%)	40 (66%)
Hospital admissions for asthma in last year		0·0 (0·0–0·0)	0·0 (0·0–0·0)
Accident and emergency department visits in last year		0·0 (0·0–0·0)	0·0 (0·0–0·0)
General practitioner visits for asthma in last year		1·0 (0·0–3·0)	1·0 (0·0–3·0)
Rescue courses of oral corticosteroids in past year		2·0 (1·0–4·0)	2·0 (1·0–4·0)
Previous admission for asthma to intensive therapy unit		55 (22·9%)	9 (14·8%)
Number of previous admissions for asthma to intensive therapy unit		1·0 (1·0–2·0)	1·0 (1·0–2·0)
Ever been ventilated		27 (11%)	4 (7%)
Rhinitis		168 (70%)	40 (66%)
Eczema		85 (35%)	15 (25%)
Nasal polyps		58 (24%)	15 (25%)
Previous nasal surgery		57 (24%)	13 (21%)
Oesophageal reflux		137 (57%)	42 (69%)
Aspirin sensitivity		33 (14%)	14 (23%)
Depression or anxiety		73 (30%)	19 (31%)
Hypertension		70 (29%)	24 (39%)
Osteoporosis or osteopenia		57 (24%)	9 (15%)
Osteoarthritis		64 (27%)	14 (23%)
Hypercholesterolaemia		39 (16%)	14 (23%)
Diabetes		28 (12%)	6 (10%)
Cataracts		26 (11%)	7 (11%)
Obstructive sleep apnoea		15 (6%)	2 (3%)
Ischaemic heart disease		8 (3%)	4 (7%)
Peptic ulcer		8 (3%)	0
Stroke		4 (2%)	2 (3%)
Chronic kidney disease		4 (2%)	3 (5%)
Glaucoma		4 (2%)	0
Myocardial infarction		2 (1%)	1 (2%)
FEV_1_		2·2 (0·7)	2·1 (0·7)
% predicted FEV_1_		75·3% (19·2)	76·5% (19·8)
Forced vital capacity		3·3 (0·9)	3·2 (0·9)
% predicted forced vital capacity		90·6% (16·3)	93·1% (18·9)
FEV_1_/forced vital capacity		0·66 (0·11)	0·65 (0·13)
PEF		380·4 (126·4)	359·6 (133·2)
Sputum eosinophils†		1·5% (0·4–9·5)	1·3% (0·2–4·3)
Sputum neutrophils		64·8% (33·5–80·4)	57·8% (30·8–79·3)
Sputum lymphocytes		0·4% (0·0–1·5)	0·5% (0·0–1·5)
Sputum macrophage		22·1% (10·6–40·1)	27·1 (10·3–49·0)
FENO, ppb		21 (13–29)	19 (12–28)
Blood eosinophil count, 10^9^ cells per L		0·20 (0·11–0·32)	0·26 (0·15–0·40)
Periostin, ng/mL		52·8 (15·7)	53·5 (18·2)
Composite biomarker score			
	0	55 (23%)	13 (21%)
	1	135 (56%)	37 (61%)
	2	48 (20%)	10 (16%)
Maintenance oral corticosteroids user		87 (36%)	24 (39%)
Oral corticosteroid dose (mg)		10 (5–10)	9 (5–10)
Inhaled oral corticosteroids dose (beclometasone dipropionate μg equivalent)		2000 (2000–2000)	2000 (2000–2000)
LAMA user		109 (45%)	35 (57%)
Patient reported outcomes			
	Asthma Control Questionnaire-7 score	2·0 (1·1)	2·0 (1·3)
	Asthma Quality of Life Questionnaire total score	4·9 (1·3)	4·7 (1·6)

**Da**ta are n (%), median (IQR), or mean (SD). LAMA=long-acting muscarinic antagonist. FENO=fractional exhaled nitric oxide. PEF=peak exploratory flow rate. ppb=parts per billion.

* Ethnicity as per Global Lung Initiative 2012.

† Sputum data was available in 123 subjects at baseline.

**Figure 2:**
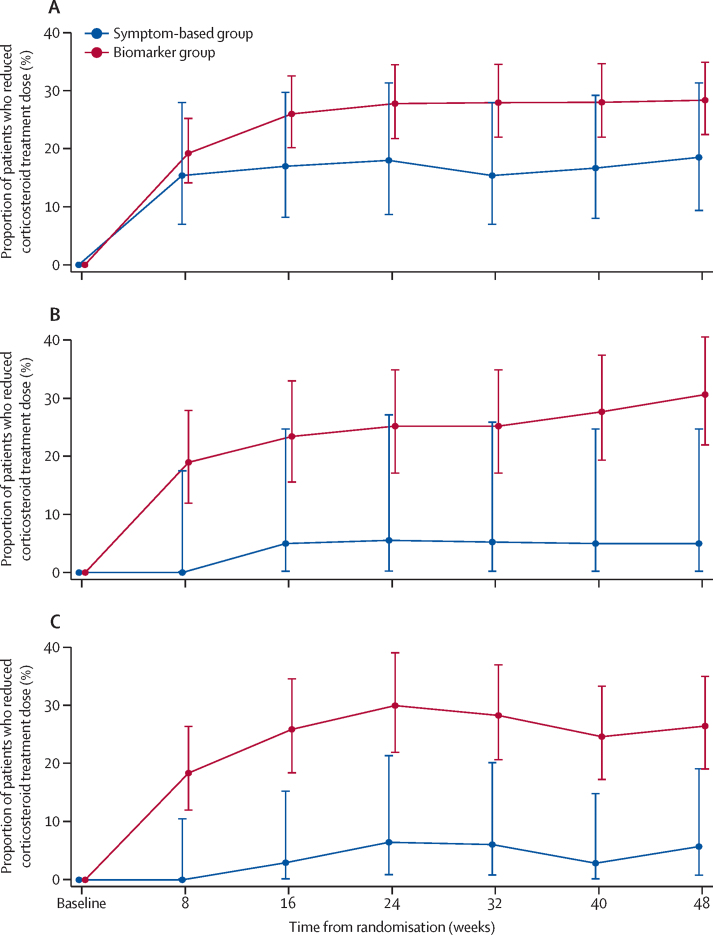
Proportion of patients who reduced corticosteroid treatment dose over 48 weeks (A) In the intention-to-treat population. (B) In the per-protocol population. (C) In the group with uncontrolled asthma at baseline (Asthma Control Questionnaire-7 ≥1·5).

For the primary outcome in the ITT population, 28·4% of patients in the biomarker strategy group reduced their dose of corticosteroid at 48 weeks compared with 18·5% of patients in the control group (adjusted odds ratio [aOR] 1·71 [95% CI 0·80–3·63]; p=0·17; figure 2A; table 4). Our estimates were similar when doing a complete-case analysis (aOR 1·70 [0·79–3·67]), and were robust to missing-not-at-random assumptions under both the best-case scenario (aOR 1·74 [0·81–3·74]) and worst-case scenario (aOR 1·69 [0·78–3·65]). There was no significant difference in secondary outcomes between study groups in the ITT analysis (table 4) and no difference in lung function, symptom scores, quality of life, or biomarkers in patients who reduced their corticosteroid dose (appendix p 9).

**Table 4:** Effects on corticosteroid treatment dose, lung function, asthma symptoms, asthma related quality of life, and type-2 biomarkers

	**Control group**		**Biomarker strategy group**		**Unadjusted analysis**		**Adjusted analysis**	
	n	Effect estimate (95% CI)	n	Effect estimate (95% CI)	Odds ratio (95% CI)	p value	Odds ratio (95% CI)	p value
**Intention-to-treat analysis**								
Decrease in corticosteroid dose*	54	18·5% (9·3 to 31·4)	208	28·4% (22·3 to 35·0)	1·73 (0·83 to 3·61)	0·14	1·71 (0·80 to 3·63)	0·17
Inhaled corticosteroid dose (beclometasone dipropionate, μg equivalent)	54	2011 (1893 to 2129)	208	1955 (1820 to 2090)	−56 (−325 to 213)	0·68	−96 (−287 to 94)	0·32
Cumulative inhaled corticosteroid dose (beclometasone dipropionate, μg equivalent)	54	724 763 (684 245 to 765 281)	209	718 298 (676 416 to 760 180)	−6465 (−90 905 to 77 976)	0·88	−4348 (−87 909 to 79 212)	0·92
Oral corticosteroid dose, mg	54	6 (4 to 8)	208	5 (4 to 6)	−1 (−3 to 2)	0·58	−1 (−2 to 1)	0·46
Cumulative oral corticosteroid dose, mg	54	1626 (1109 to 2142)	209	1631 (1327 to 1935)	6 (−643 to 654)	0·99	55 (−581 to 691)	0·87
Oral corticosteroid use*	54	55·6% (41·4 to 69·1)	208	49·5% (42·5 to 56·5)	0·78 (0·43 to 1·43)	0·43	0·79 (0·36 to 1·74)	0·56
ACQ-7 score	54	2·2 (1·9 to 2·6)	206	2·0 (1·8 to 2·1)	−0·3 (−0·6 to 0·1)	0·16	−0·1 (−0·3 to 0·2)	0·47
AQLQ total score	52	4·7 (4·2 to 5·1)	206	5·0 (4·8 to 5·2)	0·4 (−0·1 to 0·8)	0·108	0·2 (−0·2 to 0·5)	0·36
% predicted FEV_1_	54	72·1 (66·5 to 77·7)	204	73·2 (70·6 to 75·8)	1·1 (−4·7 to 6·8)	0·72	0·4 (−2·7 to 3·6)	0·80
FENO†	54	19·5 (17·5 to 25·0)	205	22·0 (19·0 to 25·0)	1·06 (0·88 to 1·29)	0·53	1·02 (0·87 to 1·19)	0·85
Blood eosinophil count, 10^9^ cells per L†	51	0·31 (0·26 to 0·39)	206	0·20 (0·18 to 0·23)	0·66 (0·49 to 0·89)	0·0062	0·79 (0·61 to 1·03)	0·078
Periostin, ng/mL	51	54·2 (48·7 to 59·7)	201	51·4 (49·5 to 53·4)	−2·8 (−7·5 to 2·0)	0·25	−2·0 (−5·0 to 1·0)	0·19
Annual exacerbation rate‡	54	1·67 (1·35 to 2·06)	208	1·36 (1·21 to 1·54)	0·82 (0·58 to 1·16)	0·26	0·84 (0·61 to 1·15)	0·28
Time to first exacerbation§	..	..	..	..	0·72 (0·50 to 1·05)	0·085	0·77 (0·53 to 1·13)	0·18
Annual hospitalisation rate‡	54	0·16 (0·08 to 0·32)	208	0·10 (0·06 to 0·15)	0·62 (0·25 to 1·50)	0·29	0·77 (0·32 to 1·84)	0·56
Refused to initiate oral corticosteroid*	16	43·8% (19·8 to 70·1)	101	56·4% (46·2 to 66·3)	1·67 (0·58 to 4·82)	0·35	0·80 (0·23 to 2·76)	0·72
**Per-protocol analysis**								
Decrease in corticosteroid dose*	20	5·0% (0·1 to 24·9)	101	30·7% (21·9 to 40·7)	8·41 (1·08 to 65·68)	0·042	11·48 (1·35 to 97·83)	0·026
Inhaled corticosteroid dose (beclometasone dipropionate μg equivalent)	20	1990 (1814 to 2166)	101	1825 (1680 to 1970)	−165 (−497 to 166)	0·33	−210 (−480 to 61)	0·13
Cumulative inhaled corticosteroid dose (beclometasone dipropionate μg equivalent)	20	706 610 (650 247 to 762 973)	101	665 711 (620 861 to 710 561)	−40 899 (−143 437 to 61 639)	0·43	−52 599 (−162 431 to 57 234)	0·35
Oral corticosteroid dose, mg	20	7 (3–10)	101	5 (4–7)	−1 (−5 to 2)	0·39	−2 (−5 to 0)	0·088
Cumulative oral corticosteroid dose, mg	20	1721 (812 to 2629)	101	1644 (1217 to 2071)	−77 (−1099 to 945)	0·88	13 (−1070 to 1096)	0·98
Oral corticosteroid use*	20	65·0% (40·8 to 84·6)	101	53·5% (43·3 to 63·5)	0·62 (0·23 to 1·68)	0·35	0·43 (0·10 to 1·78)	0·24
ACQ-7 score	20	2·4 (1·9 to 3·0)	100	1·9 (1·7 to 2·2)	−0·5 (−1·0 to 0·0)	0·071	0·1 (−0·3 to 0·5)	0·52
AQLQ total score	18	4·2 (3·5 to 4·9)	99	5·1 (4·8 to 5·4)	0·9 (0·2 to 1·6)	0·010	0·5 (−0·1 to 1·1)	0·11
% predicted FEV_1_	20	70·2 (59·0 to 81·4)	99	72·2 (68·0 to 76·5)	2·0 (−8·4 to 12·5)	0·71	−0·9 (−6·8 to 5·0)	0·76
FENO†	20	13·5 (9·0 to 25·0)	100	21·0 (19·0 to 24·0)	1·50 (1·14 to 1·97)	0·0037	1·39 (1·07 to 1·80)	0·014
Blood eosinophil count, 10^9^ cells per L†	19	0·35 (0·26 to 0·47)	100	0·19 (0·16 to 0·21)	0·63 (0·39 to 1·01)	0·053	0·95 (0·62 to 1·47)	0·83
Periostin, ng/mL	19	49·1 (44·5 to 53·7)	97	50·7 (48·2 to 53·1)	1·5 (−4·3 to 7·4)	0·61	−0·8 (−4·9 to 3·3)	0·71
Annual exacerbation rate‡	20	1·29 (0·86 to 1·92)	101	1·04 (0·86 to 1·27)	0·82 (0·45 to 1·48)	0·50	0·89 (0·49 to 1·61)	0·69
Time to first exacerbation§	..	..	..	..	0·86 (0·46 to 1·60)	0·63	0·80 (0·40 to 1·61)	0·54
Annual hospitalisation rate‡	20	0·11 (0·03 to 0·43)	101	0·12 (0·06 to 0·21)	1·09 (0·23 to 5·13)	0·91	2·15 (0·38 to 12·27)	0·39
Refused to initiate oral corticosteroids*	3	0·0% (0·0 to 70·8)	..	0·0% (0·0 to 18·5)	..	..	..	..
**Uncontrolled asthma (ACQ-7 ≥1·5)**								
Decrease in corticosteroid dose*	35	5·7% (0·7 to 19·2)	125	26·4% (18·9 to 35·0)	5·92 (1·35 to 26·04)	0·019	5·78 (1·27 to 26·23)	0·023
Inhaled corticosteroid dose (beclometasone dipropionate μg equivalent)	35	2137 (2004 to 2270)	125	2041 (1860 to 2223)	−96 (−443 to 251)	0·59	−280 (−488 to −72)	0·0084
Cumulative inhaled corticosteroid dose (beclometasone dipropionate μg equivalent)	35	746 503 (701 511 to 791 494)	126	742 762 (684 624 to 800 901)	−3741 (−115 557 to 108 076)	0·95	8525 (−103 243 to 120 292)	0·88
Oral corticosteroid dose, mg	35	7 (4 to 9)	125	6 (5 to 8)	−0 (−3 to 3)	0·76	−2 (−4 to 1)	0·15
Cumulative oral corticosteroid dose, mg	35	1864 (1159 to 2570)	126	1927 (1487 to 2366)	63 (−838 to 963)	0·89	39 (−890 to 968)	0·93
Oral corticosteroid use*	35	62·9% (44·9 to 78·5)	125	55·2% (46·0 to 64·1)	0·73 (0·34 to 1·57)	0·42	0·49 (0·17 to 1·36)	0·17
ACQ-7 score	35	2·7 (2·3 to 3·1)	123	2·5 (2·3 to 2·7)	−0·2 (−0·7 to 0·2)	0·28	0·0 (−0·3 to 0·4)	0·82
AQLQ total score	34	4·0 (3·5 to 4·5)	123	4·4 (4·1 to 4·6)	0·4 (−0·2 to 0·9)	0·17	0·2 (−0·3 to 0·7)	0·42
% predicted FEV_1_	35	66·2 (59·4 to 72·9)	122	70·5 (67·1 to 73·9)	4·4 (−2·8 to 11·5)	0·24	0·8 (−2·8 to 4·3)	0·68
FeNO†	35	18·0 (12·0 to 25·0)	122	22·0 (21·0 to 26·0)	1·38 (1·10 to 1·73)	0·0057	1·24 (1·02 to 1·51)	0·032
Blood eosinophil count, 10^9^ cells per L†	33	0·27 (0·22 to 0·36)	123	0·18 (0·16 to 0·20)	0·64 (0·43 to 0·93)	0·019	0·81 (0·57 to 1·14)	0·23
Periostin (ng/mL)	32	50·7 (45·4 to 56·0)	120	50·0 (47·8 to 52·2)	−0·7 (−5·6 to 4·2)	0·78	−0·1 (−3·1 to 2·9)	0·96
Annual exacerbation rate‡	35	1·92 (1·50 to 2·46)	125	1·70 (1·48 to 1·95)	0·88 (0·60 to 1·29)	0·52	0·93 (0·65 to 1·33)	0·69
Time to first exacerbation§	..	..	..	..	0·76 (0·49 to 1·16)	0·20	0·83 (0·53 to 1·32)	0·44
Annual hospitalisation rate‡	35	0·21 (0·10 to 0·45)	125	0·13 (0·08 to 0·21)	0·60 (0·24 to 1·50)	0·27	0·67 (0·26 to 1·71)	0·40
Refused to initiate oral corticosteroid*	11	27·3% (6·0 to 61·0)	43	41·9% (27·0 to 57·9)	1·92 (0·45 to 8·26)	0·38	1·18 (0·20 to 6·94)	0·85

**Dat**a are mean (95% CI), effect estimate shown as mean difference unless otherwise stated. ACQ-7=Asthma Control Questionnaire 7. AQLQ=Asthma Quality of Life Questionnaire. FENO=fractional exhaled nitric oxide.

* Data are proportion (95% CI), effect estimate shown as odds ratio.

† Data are median (bootstrapped 95% CI), effect estimate shown as ratio of geometric means.

‡ Value shown as rate (95% CI), effect estimate shown as rate ratio.

§ Effect estimate shown as hazard ratio.

In the prespecified PP analysis of 121 patients, a significantly greater proportion of patients were on a lower dose of corticosteroid at week 48 in the biomarker study group (30·7% of patients) compared with the control group (5·0% of patients; aOR 11·48 [95% CI 1·35–97·83]; p=0·026; figure 2B; table 4). The baseline characteristics of the PP and ITT populations were similar except that in the PP population, patients in the control group were more symptomatic compared with the biomarker strategy group (mean ACQ-7 score 2·6 [SD 1·1] *vs* 2·0 [1·1]; p=0·022) and had greater impairment of AQLQ (mean total score 4·2 [SD 1·2] *vs* 5·0 [1·2]; p=0·0086; appendix p 11). The estimated number of patients needed to treat to achieve one patient on lower corticosteroid dose was four (95% CI 3–9). As with the ITT analysis, other than FENO, there was no significant difference in secondary outcomes (table 4) and no worsening of lung function, symptom scores, quality of life, or biomarkers in patients who had a reduction in corticosteroid dose (appendix p 9).

The PP population was 40% of the ITT population as a consequence of patients not following at least one treatment advisory (n=124), withdrawing from the study (n=32), or missing at least one study visit (n=18). Overall, 1041 (85%) of 1224 treatment advisories were followed in the biomarker-based group and 257 (81%) of 318 in the control group, though this was lower in both groups when a treatment change was mandated (273 [65%] of 419 in the biomarker strategy group and 77 [58%] of 133 in the control group (appendix p 13).

There was a wide variation in the number of deviations from the protocol seen at individual clinical centres (appendix p 12). The predominant reason for not following a treatment advisory was the patient's choice not to do so (appendix p 13) and adherence to treatment advice reduced as the study progressed (appendix p 13). Patients showed a reluctance to initiate regular OCS (56% in the biomarker strategy group and 44% in the control group). We also observed a reluctance to reduce corticosteroid treatment and notably, in the biomarker strategy group, these patients were more symptomatic than those who did follow a reduce advisory (ACQ-7 2·5 [SD 1·2] *vs* 1·8 [1·0]; p<0·0001). Those who were reluctant to reduce corticosteroid treatment also had lower lung function (FEV_1_ 72·4% [SD 17·6] *vs* 82·8% [19·2]; p=0·0002) with worse airflow obstruction (FEV_1_/forced vital capacity [FVC] 65% [SD 12] *vs* 70% [12]; p=0·0016). There was no difference in the biomarker profile of patients who reduced treatment and those who did not.

The odds ratio of patients reducing corticosteroid dose in the biomarker strategy group remained significant when the PP definition was relaxed to include patients who were only non-adherent to the study protocol on one (n=200; aOR 4·54 [95% CI 1·30–15·87]; p=0·018) or two (n=237; aOR 2·64 [1·04–6·72]; p=0·041) study visits or treatment advisories.

**Figure 3:**
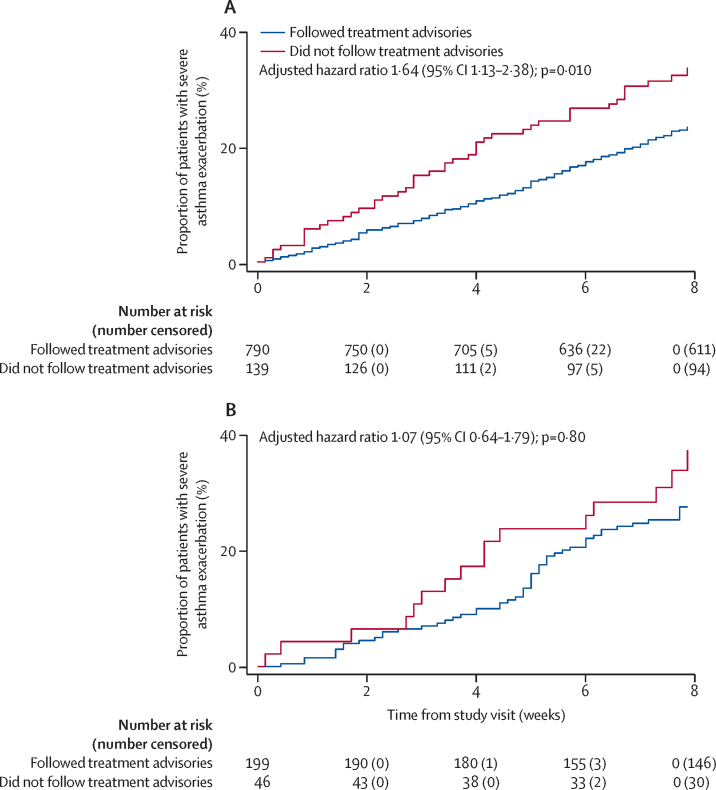
Proportion of patients who did or did not follow treatment advisories and had a severe asthma exacerbation (A) In the biomarker strategy group. (B) In the control group.

In the biomarker strategy group, exacerbation rates were significantly higher in those patients who did not follow treatment advisories compared with those who did (adjusted hazard ratio [aHR] 1·64 [95% CI 1·13–2·38]; p=0·010; figure 3A) but this was not seen in the control group (aHR 1·07 [0·64–1·79]; p=0·80; figure 3B). This increased exacerbation rate was noted in patients who did not reduce treatment as well as those who did not increase treatment in the biomarker strategy group (appendix p 14). Overall, 60% of those patients who completed the study had a protocol-defined exacerbation.

Given the greater symptom burden in patients not following advice to reduce treatment in the biomarker strategy group, we did an exploratory post-hoc analysis in those patients with uncontrolled asthma at baseline (ACQ-7 ≥1·5). These patients were predominantly female, with obesity, not in current employment owing to asthma-related ill health, reported clinical depression or anxiety, and had more unscheduled asthma care, with twice as many on maintenance OCS. They also had significantly lower FEV_1_ and FVC but no difference in FEV_1_/FVC (appendix p 15). Analysis of outcomes in this subgroup indicated that a greater proportion of patients were taking a lower dose of corticosteroid treatment at week 48 in the biomarker study group compared with management based on standard care (26·4% of patients *vs* 5·7% of patients; aOR 5·78 [95% CI 1·27–26·23]; p=0·023; figure 2C; table 4). Despite this reduction in corticosteroid, symptoms, lung function, and exacerbation rates did not change (table 4). There was also no difference in lung function, symptom scores, quality of life, or biomarker profile in patients who reduced their corticosteroid treatment (appendix p 9).

The median corticosteroid dose reduction in those patients who reduced their corticosteroid treatment in the ITT, PP, and ACQ-7 ≥1·5 analyses was 1000 μg beclometasone dipropionate equivalent consistently across all analyses (appendix p 9). There was no reduction in the median OCS dose in any analysis.

Of the 209 patients at the end of the study who felt they could identify which treatment strategy they had been assigned, 60 (36%) of 165 patients in the biomarker strategy group thought that they had been assigned to the control group, despite being told in the study information at the outset that they had a four out of five chance of being assigned to the biomarker strategy group. In the control group, 23 (52%) of 44 patients thought that they were receiving biomarker-directed treatment. There was no significant difference in whether they correctly identified their treatment group, suggesting that the strategy employed to mask patients to their treatment strategy had been effective.

An important secondary aim of the study was to identify how many patients could reduce their corticosteroid dose to the lowest dose of ICS and remain composite T2 biomarker-low. Of the patients recruited to the biomarker strategy group who completed the study, only nine (4%) of 209 patients successfully withdrew their corticosteroid treatment so that they achieved the lowest dose of ICS and remained biomarker-low (ie, a composite score of zero). Two other patients received serial advisories to reduce their corticosteroid dose because they were biomarker-low, but they did not successfully reduce to the lowest ICS dose. Taken together, 11 (5%) of 209 patients were composite biomarker-low.

There was no difference in mild or moderate adverse events between study groups (rate ratio [symptom:biomarker] 1·10, 95% CI 0·96–1·26) and there was no difference in severe adverse events between study groups (rate ratio [symptom:biomarker] 1·26, 95% CI 0·78–2·05). There was an increased rate of reported serious adverse events in the control group compared with the biomarker strategy group (rate ratio [symptom:biomarker] 1·64, 95% CI 1·04–2·60). There was no difference between study groups in the adjusted rate of hospitalisations for asthma (table 4).

## Discussion

This study compared T2 biomarker-directed corticosteroid treatment with a standardised clinical strategy based on current symptoms and recent exacerbation history in people with severe asthma treated with high-dose ICS. We found no significant difference between these strategies in the proportion of patients able to lower the dose of their corticosteroids in the ITT study population. The aim of the study was to reduce corticosteroid dose in a population of T2-biomarker-low patients and importantly, in terms of effect on the patients, there was no evidence of worsening of asthma control or changes in T2-associated biomarkers in those patients who reduced their dose.

In contrast to the findings in the ITT population, the prespecified PP analysis showed a significantly greater proportion of patients reducing their corticosteroid treatment in the biomarker strategy group compared with the control group. The larger effect size seen in the PP analysis was principally because fewer patients reduced corticosteroid treatment in the control group, which might in part have been due to patients being more symptomatic compared with the ITT control population. The predominant reason for the low number of patients in the PP control group was that patients chose not to follow treatment advisories, including (in some situations) patients not increasing treatment after loss of asthma control. In both treatment groups, as expected, patients were reluctant to initiate regular OCS, but the reluctance to reduce ICS treatment was not expected, particularly since this intention had been communicated to patients as a core aim of the study before recruitment. We noted that biomarker-low patients in the biomarker strategy group who were more symptomatic and had lower lung function were less likely to agree to reduce their corticosteroid treatment. This finding suggests that some of these patients might have benefited from a more thorough explanation of the dissociation between their symptoms and corticosteroid dose when T2 biomarkers are low, which might have assisted them in agreeing to corticosteroid reduction.

There was a broad difference between clinical centres in the proportion of participants adhering to the protocol and specifically those following treatment advisories. The reason for this is unclear and will require further research. Treatment reductions seen in the PP analysis mirrored the assumptions in our power calculations estimating the potential benefit of a biomarker-based approach.

Given that participants who chose not to reduce corticosteroid treatment in the biomarker strategy group were more symptomatic, we did a post-hoc analysis to explore the effects of biomarker-based management in patients with uncontrolled asthma (ACQ-7 ≥1·5). These findings were in line with our PP analysis, where a biomarker-directed approach to treatment resulted in a significant proportion of these symptomatic patients being able to reduce their corticosteroid treatment with no evidence of worsening in control of their asthma. As some biomarker-low patients with high symptom scores were reluctant to reduce treatment, we believe the scale of the benefit, in terms of the proportion who could reduce treatment, might have been underestimated in this analysis. As there were fewer opportunities for symptom-based treatment reduction in this sub-population, they serve as a useful treatment-stable comparator group, showing little difference in clinical outcomes in the biomarker strategy group who reduced their treatment significantly compared with the control group who did not. The uncontrolled asthma analysis is particularly relevant since it describes a common clinical problem: a highly symptomatic patient potentially overtreated with corticosteroids. By means of an asthma guideline-based approach to treatment, this group could often see their corticosteroid dose increased owing to high symptom burden, but this analysis suggests that a biomarker-based strategy in this group might successfully and substantially reduce corticosteroid treatment with no deterioration in asthma control or lung function or increased exacerbation of T2 inflammation based on biomarker profile.

This study had a high retention of patients, including those who did not follow treatment advisories, allowing the opportunity to compare the risk of severe exacerbation in participants who did and did not follow treatment advice in either study group. However, given the pragmatic nature of the study, we did not track patient adherence to treatment advisories in real time (ie, treatment adjustment was reviewed at next study visit) and it was not possible to monitor differences in the proportion of patients not following advisories at the different sites. Increased risk of exacerbation in patients with severe asthma who were biomarker-high was not unexpected but the increased risk observed in biomarker-low patients who did not reduce their corticosteroid dose was not expected. The use of both OCS and ICS has been associated with increased risk of infections and azithromycin has been shown to reduce severe exacerbations in both eosinophilic and non-eosinophilic patients.^6^, ^7^, ^17^ The NOVEL-START study showed a tendency to increased severe exacerbations in patients with mild asthma and low blood eosinophil counts (<150 cells per μL) initiating regular ICS treatment compared with treatment with as-needed salbutamol.^18^ Taken together, these data suggest that ICS in doses that suppress substantially T2 biomarkers might be harmful in some patients. If so, this makes the ability to carefully and safely titrate corticosteroid dose to T2-associated biomarker activity all the more important. However, given the difficulty that some symptomatic patients had in this study to reduce treatment when established on high-dose treatment, it might be better to use biomarkers to identify the right patient before increasing corticosteroid treatment.

The median daily ICS reduction in patients who reduced treatment was consistent across all analyses at a beclometasone dipropionate equivalent dose of 1000 μg. Our expert Delphi consensus advised that at least a 250–500 μg reduction in daily ICS treatment would be regarded as a clinically meaningful dose reduction in a severe asthma population on high-dose ICS, suggesting that severe asthma specialists will recognise this as a substantial treatment benefit.^15^

The current study also aimed to establish the prevalence of T2 biomarker-low asthma in our severe asthma population when the corticosteroid dose was reduced to a minimum, because it is recognised that exposure to corticosteroids downregulates T2 biomarkers and overtreatment might potentially overestimate the prevalence of the T2 low phenotype.^19^ At study entry, 23% of participants were composite biomarker-low, which was in line with our analysis of the placebo groups of clinical trials with lebrikizumab and omalizumab (appendix p 6).^13^ However, only 11 (5%) of 209 patients managed either to down-titrate successfully to the lowest dose of ICS and remained biomarker-low or remained serially biomarker-low and did not reduce their corticosteroid treatment. These data suggest that ICS can be reduced cautiously to relatively low maintenance levels in T2 biomarker-low patients, and this would seem important before assigning any phenotypic label. It further implies that T2 biomarker-low severe asthma, as defined in this study, is uncommon. We propose that detailed clinical assessment including corticosteroid withdrawal is important before investigational research studies and testing of clinical interventions in this T2 biomarker-low population.^20^

In summary, this study did not meet its primary outcome of showing a greater proportion of patients on a lower corticosteroid dose by means of a biomarker-based strategy in severe asthma compared with corticosteroid adjustment based on symptoms and previous exacerbation history in the ITT population. We noted a large number of patients who did not follow treatment advisories to reduce corticosteroid treatment in both treatment groups; understanding the reasons for this finding is an important area for future research. In the study population who followed treatment advice per protocol or who had uncontrolled asthma at study entry, meaningful corticosteroid reduction was achieved. A biomarker-adjusted corticosteroid strategy seems particularly beneficial in those patients where symptoms and T2 biomarker profile are discordant and, as seen in up to 50% of patients with severe asthma;^8^ we suggest that before progression to high-dose inhaled and systemic corticosteroid treatment, predictive biomarkers of therapeutic response should be assessed. Once established on this dose, biomarker-driven dose reduction can be particularly challenging in patients with high levels of asthma symptoms.

## Data sharing

Following publication of the primary and secondary analyses, individual de-identified patient data, including the data dictionary, will be made available via our data sharing portal eTRIKS indefinitely delivered via Imperial College. This will allow for maximum use of the data to improve patient care and advance medical knowledge. The trial protocol and statistical analysis plan will also be made available.
