# Correction to *Lancet Respir Med* 2021; 9: 57–68

**DOI:** 10.1016/S2213-2600(20)30601-9

**Published:** 2021-02

*Heaney LG, Busby J, Hanratty CE, et al. Composite type-2 biomarker strategy versus a symptom–risk-based algorithm to adjust corticosteroid dose in patients with severe asthma: a multicentre, single-blind, parallel group, randomised controlled trial*. Lancet Respir Med *2020*; **9:** 57–68—In this Article, in table 1, column “1”, the threshold ranges quoted should read “≥15–29”, “≥150–299”, and “≥45–54”, and in column “2”, the ranges should read “≥30”, “≥300”, and “≥55”. These corrections have been made to the online version as of Jan 4, 2021 and will be made to the printed version.

