# Supplementary material for: Composite type-2 biomarker strategy versus a symptom–risk-based algorithm to adjust corticosteroid dose in patients with severe asthma: a multicentre, single-blind, parallel group, randomised controlled trial
Source: Lancet Respir Med. 2021 Jan;9(1):57–68. doi: 10.1016/S2213-2600(20)30397-0 (PMC7783382; doi:10.1016/S2213-2600(20)30397-0)
Supplement: Supplementary appendix [file mmc1.pdf]

THE LANCET
Respiratory Medicine

Supplementary appendix

This appendix formed part of the original submission and has been peer reviewed. We post it as supplied by the authors.

Supplement to: Heaney LG, Busby J, Hanratty CE, et al. Composite type-2 biomarker strategy versus a symptom–risk-based algorithm to adjust corticosteroid dose in patients with severe asthma: a multicentre, single-blind, parallel group, randomised controlled trial. *Lancet Respir Med* 2020; published online Sept 8. http://dx.doi.org/10.1016/S2213-2600(20)30397-0.

SUPPLEMENTARY APPENDIX

Supplement to: Heaney LG, Busby J, Hanratty CE, et al. A randomised trial of treatment optimisation in patients with severe asthma using composite type-2 biomarkers to adjust corticosteroid dose versus a symptom/risk-based algorithm.

TABLE OF CONTENTS

PAGE

1. LIST OF PARTICIPATING CLINICAL CENTRES 2

2. LIST OF INVESTIGATORS AND CONTRIBUTORS 2
   2.1. List of industrial partners in RASP-UK consortium 4

3. METHODOLOGY 5
   3.1. Study subjects inclusion and exclusion criteria 5
      3.1.1. Inclusion criteria 5
      3.1.2. Exclusion criteria 5
   3.2. Generation of the composite biomarker scoring system 6
   3.3. Treatment adjustment in study arms 6

4. RESULTS 9

5. ADVERSE EVENTS 17

6. REFERENCES 18

Abbreviations

ACQ	Asthma control questionnaire
BDP	Beclometasone dipropionate
BMI	Body mass index
FeNO	Fractional exhaled nitric oxide
FEV1	Forced expiratory volume in 1 second
FP	Fluticasone propionate
FVC	Forced vital capacity
ICS	Inhaled corticosteroid
ITU	Intensive treatment unit
LABA	Long acting beta 2 agonist
LAMA	Long-acting muscarinic antagonists
MDI	Metered dose inhaler
OCS	Oral corticosteroid
PEFR	Peak expiratory flow rate

1. LIST OF PARTICIPATING CLINICAL CENTRES

NHS Clinical Centres with a dedicated tertiary care in difficult asthma service that recruited to the study

- Belfast Health & Social Care Trust
- Oxford University Hospitals NHS Trust
- Glenfield Hospital, University Hospitals of Leicester NHS Trust
- Wythenshawe Hospital, University Hospitals of South Manchester NHS Trust
- University Hospital Southampton NHS Foundation Trust
- Royal Brompton & Harefield NHS Foundation Hospital
- King's College Hospital NHS Foundation Trust
- Nottingham University Hospitals NHS Foundation Trust
- Sheffield Teaching Hospitals NHS Foundation Trust
- Gartnavel and Stobhill/Glasgow Royal Infirmary Hospitals, Greater Glasgow Health Board
- Heartlands Hospital, University Hospitals Birmingham NHS Foundation Trust
- Freemans Hospital, Newcastle upon Tyne NHS Foundation Trust

2. LIST OF INVESTIGATORS AND CONTRIBUTORS

**SITE**	
Centre for Experimental Medicine, School of Medicine, Dentistry and Biomedical Sciences, Queen's University, Belfast, UK	Prof LG Heaney MD<br>Dr J Busby PhD<br>Dr CE Hanratty PhD<br>Ms R Stone RCN
23andMe, Sunnyvale, California, USA	JG Matthews MD
Oxford Respiratory NIHR BRC, Nuffield Department of Medicine, The University of Oxford, Oxford, UK	Prof ID Pavord FMedSci<br>Ms C Borg, BA<br>Ms C Connolly, BNS
Genentech Inc., South San Francisco, California, USA	Dr JR Arron MD<br>Dr CTJ Holweg PhD<br>Mr DF Choy BS
Department of Respiratory Sciences, Institute for Lung Health and Leicester NIHR Biomedical Research Centre, University of Leicester, Leicester, UK	Prof P Bradding DM<br>Prof CE Brightling PhD<br>Ms P McCourt RN<br>Ms B Hargadon BSc
NHS Greater Glasgow and Clyde Health Board, Gartnavel Hospital and University of Glasgow (RC), Glasgow, UK	Prof R Chaudhuri MD<br>F Yang MRCP<br>Ms T Grandison BSc
NHS Greater Glasgow and Clyde, Stobhill Hospital, Glasgow, UK	Dr DC Cowan MD<br>Ms F Steffensen BSc
Clinical and Experimental Sciences, University of Southampton and NIHR Southampton Biomedical Research Centre, Southampton, UK	Prof PH Howarth MD
Division of Infection, Immunity and Respiratory Medicine, School of Biological Sciences, The University of Manchester; Manchester Academic Health Science Centre and NIHR Manchester Biomedical Research Centre, Manchester University Hospitals NHS Foundation Trust, Manchester, UK	Prof A Woodcock MD<br>Dr SJ Fowler MD<br>Dr R Niven MD<br>Ms S Leech
Niche Science & Technology Unit 26, Falstaff House, Bardolph Road, Richmond TW9 2LH, UK	Dr TC Hardman PhD<br>Dr A Horn PhD<br>Mrs G Gainsborough BSc
Nottingham Respiratory NIHR Biomedical Research Centre, University of Nottingham, Nottingham, UK	Prof TW Harrison MD<br>Prof D Shaw MD<br>Ms K Smith MA<br>Ms H Lee MSc
Clinical and Experimental Sciences, University of Southampton, NIHR Southampton Biomedical Research Centre, Southampton, UK	Prof R Djukanovic MD<br>Prof P Howarth MD<br>Dr A Azim MD
The Newcastle upon Tyne NHS Foundation Trust, Newcastle upon Tyne, UK	Dr J Lordan MD<br>Ms G Jones RSN<br>Mr G Davies BSc

University of Birmingham and Heartlands Hospital, University Hospitals Birmingham NHS Foundation Trust, Birmingham, UK	Dr AH Mansur PhD<br>M Bellamy RSN<br>Dr S Davies MD
Royal Brompton & Harefield NHS Foundation Trust, London, UK	Prof A Menzies-Gow MD<br>Ms D John<br>Ms M Nunez
University College Hospitals NHS Foundation Trust, London, UK	Prof DS Robinson MD<br>Mr J Solis
Asthma UK & British Lung Foundation Partnership, 18 Mansell Street, London, UK	Dr SM Walker PhD<br>Ms V Hudson
Guy's Severe Asthma Centre, Guy's Hospital, Guy's & St. Thomas' NHS Foundation Trust, London, UK	Dr C Corrigan MD<br>Dr D Jackson MD
National Heart and Lung Institute,<br>Faculty of Medicine, National Heart & Lung Institute, Imperial College, London	I Adcock,<br>KF Chung<br>S Johnston
Department of Medicine,<br>Royal College of Surgeons of Ireland	RW Costello
Names that are underlined are those of identified authors. Names not underlined are members of the RASP-UK consortium whose names should be shown un PubMed	

Members of the RASP-UK consortium and collaborators whose names should be listed on PubMed

[First names]	[Middle initial]	[Surname]
Ian	M	Adcock
Adnam		Azim
Mary		Bellamy
Catherine		Borg
Michelle		Bourne
Clare		Connolly
Richard	W	Costello
Chris	J	Corrigan
Sarah		Davies
Gareth		Davies
Kian	F	Chung
Gabrielle		Gainsborough
Traceyanne		Grandison
Beverley		Hargadon
Avril		Horn
Val		Hudson
David	J	Jackson
Sebastian	L	Johnston
Geraldine		Jones
Paula		McCourt
Maria		Nunez
Dominic	E	Shaw
Katherine		Smith
Joel		Solis
Roisin		Stone
Freda		Yang

2.1. List of industrial partners in RASP-UK consortium

GSK
Hoffman la Roche / Genentech Inc
Amgen
Astra Zeneca / Medimmune
Boehringer Ingelheim
Jannsen
Circassia
Vitalograph

3. METHODOLOGY

3.1. Study subjects inclusion and exclusion criteria

The full study protocol has been previously published [1].

3.1.1. Inclusion criteria

Patients must meet the following criteria at screening for study entry (patients can be rescreened for study entry up to three times):

1. Age ≥ 18 and ≤ 80 years at screening visit;
2. Able and willing to provide written informed consent and to comply with the study protocol;
3. Baseline fractional exhaled nitric oxide (FeNO) < 45 ppb at screening;
4. Severe asthma confirmed after assessment by an asthma specialist. Diagnosed with asthma at least 12 months prior to screening;
5. Current asthma treatment with long acting beta 2 agonist (LABA) plus high doses of inhaled corticosteroids (≥1000 µg fluticasone propionate (FP) daily or equivalent);
6. Patients on an inhaled corticosteroid (ICS)/LABA single inhaler strategy must be switched to fixed dosing ICS/LABA for 4 weeks prior to screening;
7. Documented history of reversibility of ≥12% change in forced expiratory volume in 1 second (FEV1) within the past 24 months or during screening period, as demonstrated by:

   Documented airflow obstruction (FEV1/forced vital capacity (FVC) <70%), where FEV1 has varied by ≥12% either spontaneously or in response to oral corticosteroid (OCS) therapy or bronchodilators either between or during clinic visits

   or

   A 20% drop in FEV1 to methacholine <8 mg/mL or a 15% fall in FEV1 after inhaling a cumulative dose of mannitol of ≤635 mg indicating the presence of airway hyperresponsiveness. If sites customarily use histamine to perform tests of airway responsiveness, this may be used in place of methacholine.

3.1.2. Exclusion criteria

Patients who meet any of the following criteria will be excluded from study entry

1. Acute exacerbation requiring oral corticosteroids in previous 4 weeks before screening (subjects were eligible for rescreening and inclusion).
2. If recently commenced on a leukotriene receptor antagonist or theophylline, stable on treatment for 4 weeks prior to screening.
3. Current self-reported history of smoking (including electronic inhaled nicotine products) or former smoker with a smoking history of >15 pack-years:
   a. A current smoker is defined as someone who has smoked one or more cigarettes per day (or marijuana or pipe or cigar) for ≥ 30 days within the 24 months prior to the screening visit (Day -14) and / or cotinine positive at screening;
   b. Any individual who smokes (cigarettes, marijuana, pipe, or cigar) occasionally, even if for < 30 days within the 24 months prior to the screening visit (Day -14), must agree to abstain from all smoking from the time of consent through completion of study;
   c. A former smoker is defined as someone who has smoked one or more cigarettes per day (or marijuana or pipe or cigar) for ≥ 30 days in his or her lifetime (as long as the 30-day total did not include the 24 months prior to the screening visit [Day -14]);
   d. A pack-year is defined as the average number of packs per day times the number of years of smoking.
4. Known current malignancy or current evaluation for a potential malignancy or history of malignancy within 5 years prior to baseline. With the exception of basal-cell and squamous-cell carcinomas of the skin and carcinoma in situ of the cervix uteri that have been excised and cured.

virus infection or currently receiving or have historically received intravenous immunoglobulin for treatment for immunodeficiency.

6. Other clinically significant medical disease or uncontrolled concomitant disease despite treatment that is likely, in the opinion of the investigator, to require a change in therapy or impact the ability to participate in the study.
7. History of current alcohol, drug, or chemical abuse or past abuse that would impair or risk the subject's full participation in the study, in the opinion of the investigator.
8. Current use of an immunomodulatory/immunosuppressive therapy or past use within 3 months or five drug half-lives (whichever is longer) prior to the screening visit.
9. Use of a biologic therapy including omalizumab at any time during the 6 months prior to the screening visit.
10. Bronchial thermoplasty within prior 6 months of the screening visit.
11. Initiation of or change in allergen immunotherapy within 3 months prior to the screening visit.
12. Treatment with an investigational agent within 30 days of the screening visit (or five half lives of the investigational agent, whichever is longer).
13. Female patients who are pregnant or lactating.

3.2. Generation of the composite biomarker scoring system

The predictive value of using FeNO, blood eosinophils and periostin as a composite biomarker to predict exacerbation risk was examined in the placebo arms of clinical trials with lebrikizumab and omalizumab in patients taking at least 500 µg FP and a second controller [2,3,4].

These studies were designed to prospectively collect exacerbation events. The analysis demonstrated these biomarkers are all correlated with exacerbation risk but using the three biomarkers based on the tertile thresholds in these studies, the composite biomarker low group (FeNO <15 ppb, blood eosinophil count<150 /µL and periostin <45 ng/mL) had a 4-fold lower risk of exacerbation compared to the maximum score of 6 (FeNO >30 ppb, blood eosinophil count >300/µL and periostin >55 ng/ml). The scoring system was based on the average score of the sum of the three biomarkers (table 1, main manuscript). This composite biomarker score was independent of symptom score and the predictive value was identical in subjects on both oral corticosteroids and inhaled corticosteroids when compared to those on inhaled corticosteroids alone, allowing the scoring system to be used across the spectrum of severe asthma. In subjects with FeNO<45 ppb (n=314), in this analysis, 78 (24.8%), 187 (59.6%) and 49 (15.6%) had composite scores of 0, 1 and 2, respectively. This score was used to make a treatment advisory adjustment to corticosteroid treatment (table 1, main manuscript).

3.3. Treatment adjustment in study arms

The following treatment adjustment table was used to guide treatment changes according in the biomarker treatment arm:

.

Steroid therapy step	Seretide MDI	Seretide Accuhaler	Symbicort Turbohaler	Flutiform MDI	Relvar Ellipta	Other LABA / ICS combinations (FP equivalent dose per day)
Step 1	Seretide 50<br>2 bd	Seretide 100<br>1 bd	Symbicort 6/200 1 bd<br>If ACQ≥1.5 add OXIS 12 1 bd (or equivalent)	Flutiform 50 2 bd<br>If ACQ≥1.5 add OXIS 12 1 bd (or equivalent)	Seretide 100 Accuhaler 1 bd	LABA/FP equivalent – 200 µg per day
Step 2	Seretide 125<br>2 bd	Seretide 250<br>1 bd	Symbicort 6/200 2 bd<br>If ACQ≥1.5 add OXIS 12 1 bd (or equivalent)	Flutiform 125 2 bd<br>If ACQ≥1.5 add OXIS 12 1 bd (or equivalent)	Relvar 22/92 1 mane	LABA/FP equivalent – 500 µg per day
Step 3	Seretide 250<br>2 bd	Seretide 500<br>1 bd	Symbicort 12/400 2 bd	Flutiform 250 2x bd	Relvar 22/184 1 mane	LABA/FP equivalent – 1000 µg per day
Step 4	Seretide 250<br>2 bd µg<br>Prednisolone<br>5 mg per day	Seretide 500<br>1 bd<br>Prednisolone<br>5 mg per day	Symbicort 12/400 2 bd<br>Prednisolone 5 mg per day	Flutiform 250 2 bd<br>Prednisolone 5 mg per day	Relvar 22/184 1 mane<br>Prednisolone 5 mg per day	LABA/FP equivalent – 1000 µg per day<br>Prednisolone 5 mg per day
Step 5	Seretide 250<br>2 bd<br>Prednisolone 10 mg per day	Seretide 500<br>1 bd<br>Prednisolone<br>10 mg per day	Symbicort 12/400 2 bd<br>Prednisolone 10 mg per day	Flutiform 250 2 bd<br>Prednisolone 10 mg per day	Relvar 22/184 1 mane<br>Prednisolone 10 mg per day	LABA/FP equivalent – 1000 µg per day<br>Prednisolone 10 mg per day
Step 6	Seretide 250<br>2 bd<br>Prednisolone 15 mg per day	Seretide 500<br>1 bd plus Prednisolone 15 mg per day	Symbicort 12/400 2 bd plus<br>Prednisolone 15 mg per day	Flutiform 250 2 bd<br>Prednisolone 15 mg per day	Relvar 22/184 1 mane plus<br>Prednisolone 15 mg per day	LABA/FP equivalent – 1000 µg per day plus<br>Prednisolone 15 mg per day
Step 7*	Seretide 250<br>2 bd<br>Prednisolone 20 mg per day	Seretide 500<br>1 bd<br>Prednisolone<br>20 mg per day	Symbicort 12/400 2 bd<br>Prednisolone 20 mg per day	Flutiform 250 2 bd<br>Prednisolone 20 mg per day	Relvar 22/184 1 mane<br>Prednisolone 20 mg per day	LABA/FP equivalent – 1000 µg per day plus<br>Prednisolone 20 mg per day

[illegible] = Asthma control questionnaire; FP = fluticasone proprionate; ICS = inhaled corticosteroid; LABA = long acting beta 2 agonist (LABA); MDI = metered dose inhaler

The following treatment adjustment table was used to guide treatment changes according in the symptom based treatment arm.

**Step 1**	LABA / Low dose ICS (FP 200 µg or equivalent)
**Step 2**	LABA / Moderate dose ICS (500 µg FP equivalent)
**Step 3**	LABA / High dose ICS (1000 µg FP equivalent)
**Step 4**	Add Tiotropium
**Step 5**	Add regular oral steroids (starting dose 5 – 10 mg per day increasing in 5 mg increments)

FP = fluticasone proprionate; ICS = inhaled corticosteroid; LABA = long acting beta 2 agonist (LABA)

Treatment guidance footnotes:

- it is recognised that on some occasions patients my require higher doses of systemic steroids beyond 20 mg prednisolone per day – as with all treatment steps, particular attention should be paid to adherence with prednisolone but if required prednisolone can be increased in further 5 mg increments.
- the therapeutic adjustments are designed to reflect clinical practice and to be pragmatic and allow accommodation of currently used combination inhaler therapies in this population - because of this, ICS will be adjusted in line with the patient's prescribed inhaler LABA/ICS inhaler device. This will mean in some situations that LABA is adjusted along with ICS which would reflect usual clinical practice.
- if patients on theophylline, leukotriene receptor antagonist at baseline, these are not adjusted during study – they are not added during study.
- in symptom based arm, if patient has an ACQ7>1.5 and corticosteroid is NOT increased, t1iotropium should be added if no contraindications, if patient is not already on LAMA therapy or nebulised short-acting anti-muscarinic therapy.
- if on inhaled steroid monotherapy (nebulised or inhaled) in addition to ICA/LABA combination therapy, the inhaled steroid monotherapy will be withdrawn initially.
- if a patient is on oral steroids and reduces to 5 mg per day, they should be advised to omit their prednisolone on the morning of their next study visit – at that visit, they should have a morning cortisol checked locally as part of routine clinical care:
  - if cortisol within normal range of local laboratory reference value, steroids can be stopped completely if indicated by study algorithm;
  - if cortisol is present but outside normal reference range of local laboratory, gradual oral steroid withdrawal in 1 mg increments is carried out;
  - if cortisol is undetectable, prednisolone is maintained at 5 mg for study duration.

RESULTS

le E2: Median change in corticosteroid treatment dose, lung function, asthma symptoms, asthma related quality of life and type-2 biomarkers by treatment direction in the ITT, PP ACQ≥1.5 analyses

le E2a ITT analysis

Outcome	Biomarker (N=236)			Symptom-based (N=60)		
	Reduce (N=67)	Maintain (N=98)	Increase (N=71)	Reduce (N=11)	Maintain (N=25)	Increase (N=24)
ICS Dose (BDP µg equivalent)	-1000 (-1600, 0)	0 (0, 0)	0 (0, 0)	-1000 (-1600, 0)	0 (0, 0)	0 (0, 0)
OCS dose (mg)	0 (-5,0)	0 (0,0)	5 (5,10)	0 (-6,0)	0 (0,0)	5 (4,10)
ACQ-7 score	0.3 (-0.3,0.7)	0.0 (-0.3,0.7)	-0.3 (-0.7,0.3)	0.3 (0.0,0.7)	-0.1 (-0.6,0.1)	0.4 (-0.4,0.9)
AQLQ total score	-0.1 (-0.7,0.4)	-0.1 (-0.5,0.3)	0.2 (-0.4,0.8)	0.0 (-0.1,0.4)	0.0 (-0.4,0.4)	-0.0 (-0.9,0.8)
% Predicted FEV1	-2.4 (-8.9,1.0)	-2.0 (-6.4,3.0)	0.0 (-5.7,5.4)	-2.3 (-5.0,1.7)	-1.2 (-6.4,3.4)	-1.8 (-5.7,2.6)
FeNO (ppb)	3.0 (0.0,11.0)	3.0 (-4.0,8.0)	-2.0 (-10.0,7.0)	9.0 (4.0,13.0)	0.0 (-5.0,3.0)	0.0 (-4.5,8.0)
Blood eosinophil count (10^9 cells/L)	0.05 (-0.01,0.11)	0.01 (-0.06,0.09)	-0.11 (-0.23,0.01)	0.06 (-0.06,0.13)	0.03 (-0.04,0.10)	0.00 (-0.13,0.12)
Periostin (ng/mL)	2.5 (-1.1,6.8)	-0.3 (-4.8,3.7)	-5.5 (-13.6,0.5)	2.9 (-1.3,4.7)	1.2 (-5.2,9.6)	-2.7 (-8.8,2.6)

e E2b PP analysis

Outcome	Biomarker (N=101)			Symptom-based (N=20)		
	Reduce (N=31)	Maintain (N=38)	Increase (N=32)	Reduce (N=1)	Maintain (N=8)	Increase (N=11)
ICS Dose (BDP µg equivalent)	-1000 (-1500, 0)	0 (0, 0)	0 (0, 0)	-1000 (-1000,-1000)	0 (0, 0)	0 (0, 0)
OCS dose (mg)	0 (-5,0)	0 (0,0)	5 (5,10)	0 (0,0)	0 (0,0)	5 (1,10)
ACQ-7 score	0.3 (-0.3,0.9)	0.0 (-0.3,0.4)	-0.3 (-1.0,0.2)	0.0 (0.0,0.0)	-0.2 (-0.6,0.0)	0.1 (-0.6,0.6)
AQLQ total score	-0.1 (-0.5,0.4)	-0.1 (-0.8,0.3)	0.5 (0.1,1.0)	0.4 (0.4,0.4)	-0.0 (-0.4,0.2)	-0.1 (-0.2,0.5)
% Predicted FEV1	-2.3 (-9.6,1.1)	-2.0 (-6.1,3.3)	0.8 (-6.6,8.7)	-2.0 (-2.0,-2.0)	-0.6 (-5.7,6.3)	-0.3 (-4.6,2.5)
FeNO (ppb)	4.0 (0.0,13.0)	2.0 (-4.0,6.0)	-2.0 (-8.0,7.0)	9.0 (9.0,9.0)	-6.0 (-10.5,0.5)	-1.0 (-7.0,1.0)
Blood eosinophil count (10^9 cells/L)	0.06 (-0.03,0.14)	0.00 (-0.07,0.06)	-0.09 (-0.20,-0.01)	0.13 (0.13,0.13)	-0.05 (-0.07,0.05)	0.00 (-0.10,0.07)
Periostin (ng/mL)	2.4 (-0.4,5.9)	0.8 (-3.0,6.9)	-2.9 (-9.3,0.5)	-0.1 (-0.1,-0.1)	2.5 (-1.9,8.7)	0.8 (-9.1,4.9)

e E2c Uncontrolled patients (ACQ≥1.5)

Outcome	Biomarker (N=147)			Symptom-based (N=39)		
	Reduce (N=40)	Maintain (N=63)	Increase (N=44)	Reduce (N=2)	Maintain (N=19)	Increase (N=18)
ICS Dose (BDP µg equivalent)	-1000 (-1500, 0)	0 (0, 0)	0 (0, 0)	-600 (-1200, 0)	0 (0, 0)	0 (0, 0)
OCS dose (mg)	0 (-5,0)	0 (0,0)	5 (5,10)	-3 (-5,0)	0 (0,0)	5 (3,10)
ACQ-7 score	0.2 (-0.4,0.7)	-0.1 (-0.4,0.4)	-0.4 (-1.1,0.1)	-0.9 (-1.3,-0.4)	-0.1 (-0.6,0.3)	0.1 (-0.6,0.7)
AQLQ total score	-0.0 (-0.7,0.8)	0.1 (-0.6,0.6)	0.3 (-0.3,1.0)	0.7 (0.4,1.1)	0.1 (-0.3,0.3)	0.3 (-0.1,1.0)
% Predicted FEV1	-1.2 (-7.9,1.4)	-1.5 (-6.5,3.5)	0.8 (-5.7,9.3)	-0.3 (-2.3,1.7)	-2.8 (-7.8,3.2)	-1.8 (-4.8,2.5)
FeNO (ppb)	5.0 (0.0,12.0)	2.0 (-4.0,8.0)	-1.5 (-10.5,7.5)	10.0 (7.0,13.0)	0.0 (-3.0,8.0)	-0.5 (-5.0,4.0)
Blood eosinophil count (10^9 cells/L)	0.05 (-0.01,0.11)	-0.00 (-0.07,0.05)	-0.11 (-0.23,-0.01)	-0.04 (-0.07,-0.01)	0.00 (-0.05,0.10)	-0.06 (-0.13,0.02)
Periostin (ng/mL)	1.5 (-2.6,5.9)	-0.2 (-4.1,3.3)	-4.2 (-9.3,0.1)	-1.8 (-2.3,-1.3)	-0.2 (-5.2,3.0)	-2.7 (-9.1,2.6)

Q = Asthma control questionnaire; BDP = beclometasone dipropionate; FeNO = fractional exhaled nitric oxide; FEV1 = forced expiratory volume in 1 second; ICS = inhaled corticosteroid;
S = oral corticosteroid

Table E3a Demographics, medical history, lung function, biomarkers, corticosteroid treatment and patient reported outcomes in Per-Protocol population by treatment arm

	Biomarker	Symptom	P-value
**Number of Patients**	101	20	
**Age At Inclusion (y)**	57.9 (13.0)	54.4 (11.8)	0.257
**Gender**			0.335
Female	70 (69.3%)	16 (80.0%)	
Male	31 (30.7%)	4 (20.0%)	
**Ethnicity**[ǂ]			0.869
Caucasian	95 (94.1%)	19 (95.0%)	
Non-Caucasian	6 (5.9%)	1 (5.0%)	
**BMI (kg/m^2)**	**31.6 (6.8)**	**33.3 (6.7)**	0.302
**Smoking Status**			0.169
Never smoked	80 (79.2%)	13 (65.0%)	
Ex-smoker	21 (20.8%)	7 (35.0%)	
**Working Status**			0.585
Not working due to asthma related ill health	10 (9.9%)	4 (20.0%)	
Not working due to other cause	41 (40.6%)	7 (35.0%)	
Working part-time due to asthma related ill health	5 (5.0%)	1 (5.0%)	
Working part-time due to other cause	18 (17.8%)	1 (5.0%)	
Student	0 (0.0%)	0 (0.0%)	
Full time	27 (26.7%)	7 (35.0%)	
**Atopic disease**	65 (64.4%)	16 (80.0%)	0.191
**Hospital admissions for asthma in last year**	0.0 (0.0,0.0)	0.0 (0.0,0.0)	0.409
**A&E visits in last year**	0.0 (0.0,0.0)	0.0 (0.0,1.0)	0.276
**GP visits for asthma in last year**	1.0 (0.0,3.0)	2.5 (1.0,4.5)	0.024
**Rescue courses of oral steroids in last year**	2.0 (1.0,4.0)	3.0 (1.5,4.0)	0.305
**Prior admission for asthma to ITU**	16 (15.8%)	4 (20.0%)	0.647
**Number of prior admissions for asthma to ITU**	1.0 (1.0,1.0)	1.0 (1.0,4.5)	0.320
**Ever been ventilated**	4 (4.0%)	2 (10.0%)	0.329
**Rhinitis**	67 (66.3%)	15 (75.0%)	0.449
**Eczema**	36 (35.6%)	4 (20.0%)	0.174
**Nasal polyps**	21 (20.8%)	7 (35.0%)	0.169
**Prior nasal surgery**	22 (21.8%)	5 (25.0%)	0.752
**Gastro-oesophageal reflux**	59 (58.4%)	15 (75.0%)	0.164
**Aspirin sensitivity**	15 (14.9%)	5 (25.0%)	0.264
**Depression / anxiety**	30 (29.7%)	9 (45.0%)	0.181
**Hypertension**	36 (35.6%)	6 (30.0%)	0.628
**Osteoporosis / osteopenia**	27 (26.7%)	6 (30.0%)	0.764
**Osteoarthritis**	31 (30.7%)	5 (25.0%)	0.611
**Hypercholesterolaemia**	20 (19.8%)	5 (25.0%)	0.600
**Diabetes**	11 (10.9%)	0 (0.0%)	0.122
**Cataracts**	13 (12.9%)	0 (0.0%)	0.089
**Obstructive sleep apnoea**	5 (5.0%)	0 (0.0%)	0.310
**Ischaemic heart disease**	5 (5.0%)	1 (5.0%)	0.993
**Peptic ulcer**	4 (4.0%)	0 (0.0%)	0.365
**Stroke**	2 (2.0%)	0 (0.0%)	0.526
**Chronic kidney disease**	1 (1.0%)	0 (0.0%)	0.655
**Glaucoma**	3 (3.0%)	0 (0.0%)	0.435
**Myocardial infarction**	1 (1.0%)	1 (5.0%)	0.199
**FEV_1**	2.0 (0.7)	1.9 (0.8)	0.395
**% Predicted FEV_1**	74.1 (20.8)	71.1 (23.2)	0.568
**FVC**	3.1 (0.9)	2.9 (0.7)	0.279
**% Predicted FVC**	89.5 (18.1)	87.7 (13.4)	0.680
**FEV_1/FVC**	0.65 (0.12)	0.64 (0.17)	0.777
**PEFR (L/m)**	363.6 (120.8)	322.5 (134.4)	0.184
**Sputum eosinophils (%)***	1.4 (0.5,5.4)	1.6 (1.0,8.0)	0.694
**Sputum neutrophils (%)**	65.8 (35.0,84.1)	48.6 (19.1,63.3)	0.228
**Sputum lymphocytes (%)**	0.5 (0.0,1.5)	0.2 (0.0,0.5)	0.272
**Macrophage sputum (%)**	23.2 (9.5,41.0)	39.6 (27.1,73.0)	0.087
**FeNO (ppb)**	20 (14,29)	18 (14,23)	0.388
**Blood eosinophil count (10^9 cells/L)**	0.18 (0.11,0.28)	0.33 (0.15,0.45)	0.016
**Periostin (ng/ml)**	51.6 (13.3)	48.3 (10.7)	0.306
**Composite score**			0.882
0	20 (19.8%)	3 (15.0%)	
1	67 (66.3%)	14 (70.0%)	
2	14 (13.9%)	3 (15.0%)	
OCS User	38 (37.6%)	9 (45.0%)	0.536
OCS Dose (mg)	10 (7,10)	8 (5,10)	0.206
**ICS Dose (BDP)**	2000 (2000,2000)	2000 (2000,2000)	0.914
**LAMA User**	48 (47.5%)	14 (70.0%)	0.066
**ACQ-7 Score**	2.0 (1.1)	2.6 (1.1)	0.022
**AQLQ Total Score**	5.0 (1.2)	4.2 (1.2)	0.009

* Sputum data was available in 48 subjects at baseline; [ǂ] Ethnicity as per Global Lung Initiative

ACQ = Asthma control questionnaire; BDP = beclometasone dipropionate; BMI = Body mass index; FeNO = fractional exhaled nitric oxide; FEV1 = forced expiratory volume in 1 second; FVC = Forced vital capacity; ICS = inhaled corticosteroid; ITU = intensive treatment unit; LAMA = long-acting muscarinic antagonists; OCS = oral corticosteroid

Figure E1 Percentage of subjects at individual sites adhering to study protocol and contributing to PP analysis

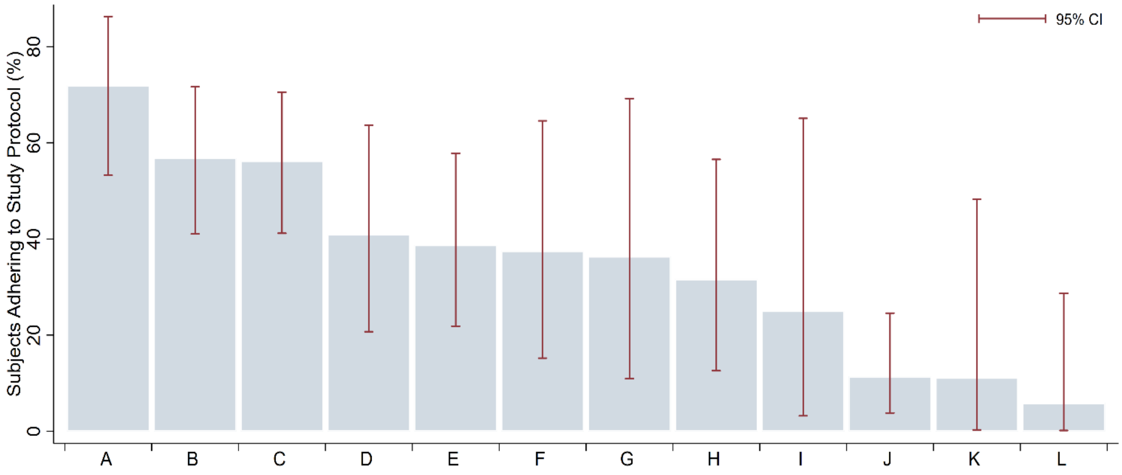

Table E4: Treatment advisories followed in each study arm (a) by number of advisories and (b) by number of patients

Advisory	Biomarker	Symptom
Reduce treatment	134 of 202 (66.3%)	30 of 53 (56.6%)
Maintain treatment	768 of 805 (95.4%)	180 of 185 (97.3%)
Increase treatment	139 of 217 (64.1%)	47 of 80 (58.8%)

Excludes omission of reduce advisories pre-defined within protocol (patient had reached the lowest permitted dose of ICS or patient had evidence of the hypothalamic pituitary adrenal axis suppression on 5 mg prednisolone)

Table E5 Reasons for patients not adjusting treatment after an advisory

**Reduce Treatment**	**N (%)**		**Increase Treatment**	**N (%)**
Patient choice	47 (51.6%)		Patient choice	84 (75.7%)
Logistical error	10 (11.0%)		Logistical error	9 (8.1%)
Asthma control deteriorated	10 (11.0%)		Asthma control deteriorated	2 (1.8%)
Exacerbation	8 (8.8%)		Exacerbation	1 (0.9%)
Clinician Decision	8 (8.8%)		Clinician Decision	8 (7.2%)
Patient error	4 (4.4%)		Patient error	4 (3.6%)
Unclear	4 (4.4%)		Unclear	1 (0.9%)
			GP Decision	2 (1.8%)

Table E6 Patient following of advisories by study visit

Visit	**Biomarker**	**Symptom**
**Baseline (0 Wks)**	212 (91.4%)	56 (93.3%)
**Visit 1 (8 Wks)**	175 (82.2%)	45 (83.3%)
**Visit 2 (16 Wks)**	173 (83.6%)	40 (75.5%)
**Visit 3 (24 Wks)**	159 (82.8%)	39 (79.6%)
**Visit 4 (32 Wks)**	157 (81.8%)	37 (74.0%)
**Visit 5 (40 Wks)**	165 (87.8%)	40 (76.9%)

Figure E2 Exacerbation rate in subjects not following treatment advisories in both study arms Biomarker Arm

Biomarker Arm

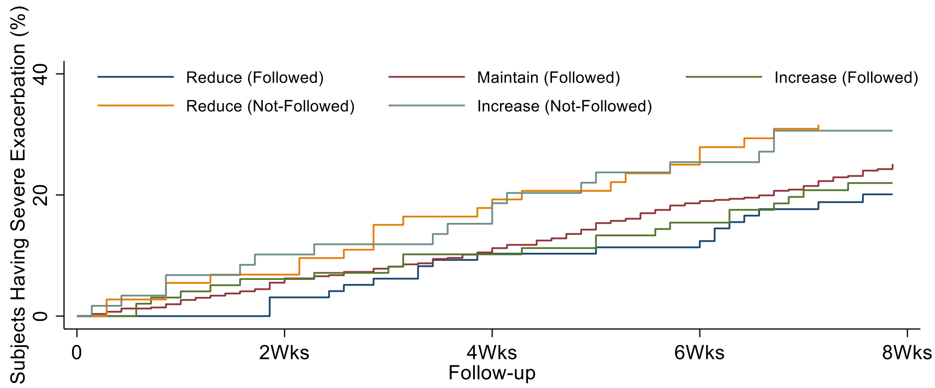

Number at Risk					
Reduce (Followed)	97	94	87	85	0
Maintain (Followed)	562	531	499	442	0
Increase (Followed)	98	92	87	80	0
Reduce (Not-Followed)	73	68	58	52	0
Increase (Not-Followed)	59	53	50	43	0

Symptom-based Arm

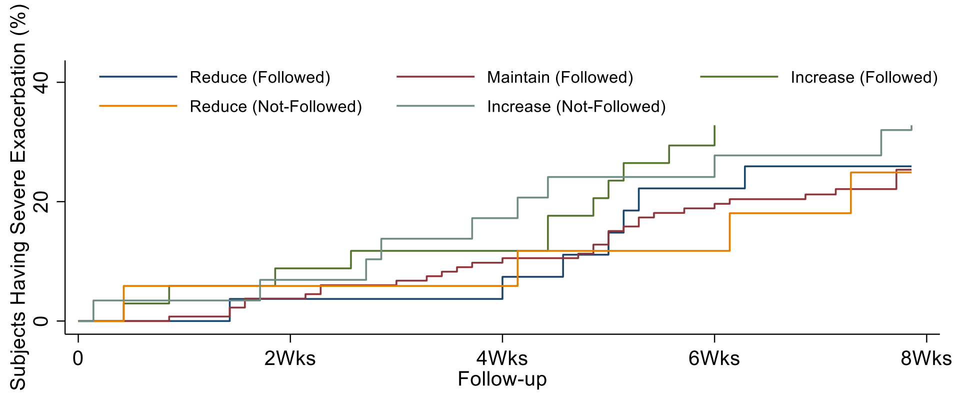

Number at Risk					
Reduce (Followed)	27	26	26	21	0
Maintain (Followed)	133	128	119	106	0
Increase (Followed)	34	31	30	24	0
Reduce (Not-Followed)	17	16	16	14	0
Increase (Not-Followed)	29	27	24	21	0

Table E7a: Demographics, medical history, lung function, biomarkers, corticosteroid treatment and patient reported outcomes in the ACQ≥1.5 Population

	ACQ<1.5	ACQ≥1.5	P-value
**Number of Patients**	113	188	
**Age At Inclusion (y)**	57.3 (14.0)	54.7 (12.5)	0.095
**Gender**			0.003
Female	61 (54.0%)	133 (70.7%)	
Male	52 (46.0%)	55 (29.3%)	
**Ethnicity** [†]			0.565
Caucasian	106 (93.8%)	173 (92.0%)	
Non-Caucasian	7 (6.2%)	15 (8.0%)	
**BMI (kg/m^2)**	28.9 (6.1)	33.3 (7.3)	<0.001
**Smoking status**			0.980
Never smoked	84 (74.3%)	140 (74.5%)	
Ex-smoker	29 (25.7%)	48 (25.5%)	
**Working status**			<0.001
Not working due to asthma related ill health	4 (3.5%)	48 (25.5%)	
Not working due to other cause	50 (44.2%)	52 (27.7%)	
Working part-time due to asthma related ill health	3 (2.7%)	10 (5.3%)	
Working part-time due to other cause	23 (20.4%)	16 (8.5%)	
Student	0 (0.0%)	2 (1.1%)	
Full time	33 (29.2%)	59 (31.4%)	
**Atopic disease**	80 (70.8%)	127 (67.6%)	0.601
**Hospital admissions for asthma in last year**	0.0 (0.0,0.0)	0.0 (0.0,0.0)	0.227
**A&E visits in last year**	0.0 (0.0,0.0)	0.0 (0.0,1.0)	0.018
**GP visits for asthma in last year**	0.0 (0.0,1.0)	2.0 (0.0,4.0)	<0.001
**Rescue courses of oral steroids in last year**	1.0 (0.0,3.0)	3.0 (1.0,4.0)	<0.001
**Prior admission for asthma to ITU**	17 (15.0%)	47 (25.0%)	0.041
**Number of prior admissions for asthma to ITU**	1.0 (1.0,1.0)	1.0 (1.0,3.0)	0.032
**Ever been ventilated**	6 (5.3%)	25 (13.3%)	0.179
**Rhinitis**	80 (70.8%)	128 (68.1%)	0.622
**Eczema**	34 (30.1%)	66 (35.1%)	0.371
**Nasal polyps**	29 (25.7%)	44 (23.4%)	0.658
**Prior nasal surgery**	27 (23.9%)	43 (22.9%)	0.839
**Oesophageal reflux**	53 (46.9%)	126 (67.0%)	<0.001
**Aspirin sensitivity**	14 (12.4%)	33 (17.6%)	0.232
**Depression / anxiety**	17 (15.0%)	75 (39.9%)	<0.001
**Hypertension**	29 (25.7%)	65 (34.6%)	0.106
**Osteoporosis / osteopenia**	13 (11.5%)	53 (28.2%)	<0.001
**Osteoarthritis**	25 (22.1%)	53 (28.2%)	0.245
**Hypercholesterolaemia**	17 (15.0%)	36 (19.1%)	0.365
**Diabetes**	10 (8.8%)	24 (12.8%)	0.299
**Cataracts**	14 (12.4%)	19 (10.1%)	0.539
**Obstructive sleep apnoea**	4 (3.5%)	13 (6.9%)	0.219
**Ischaemic heart disease**	5 (4.4%)	7 (3.7%)	0.763
**Peptic ulcer**	3 (2.7%)	5 (2.7%)	0.998
**Stroke**	2 (1.8%)	4 (2.1%)	0.830
**Chronic kidney disease**	2 (1.8%)	5 (2.7%)	0.620
**Glaucoma**	1 (0.9%)	3 (1.6%)	0.602
**Myocardial infarction**	1 (0.9%)	2 (1.1%)	0.880
**FEV_1**	2.4 (0.7)	2.0 (0.7)	<0.001
**% predicted FEV_1**	83.6 (17.6)	70.7 (18.6)	<0.001
**FVC**	3.6 (0.9)	3.0 (0.8)	<0.001
**% predicted FVC**	99.2 (15.1)	86.2 (16.0)	<0.001
**FEV_1/FVC**	0.66 (0.11)	0.65 (0.12)	0.345
**PEFR (L/m)**	430.2 (132.9)	344.2 (113.6)	<0.001
**Sputum eosinophils (%)***	2.3 (0.5,16.7)	1.1 (0.3,4.3)	0.055
**Sputum neutrophils (%)**	53.0 (27.3,78.0)	67.4 (43.0,79.6)	0.146
**Sputum lymphocytes (%)**	0.3 (0.0,1.7)	0.5 (0.0,1.3)	0.698
**Macrophage Sputum (%)**	24.1 (10.3,41.0)	23.5 (11.8,39.8)	0.955
**FeNO (ppb)**	22 (14,29)	19 (13,28)	0.314
**Blood eosinophil count (10^9 cells/L)**	0.22 (0.11,0.36)	0.20 (0.10,0.32)	0.144
**Periostin (mg/ml)**	54.9 (18.0)	51.7 (15.0)	0.094
**Composite score**			0.350
0	22 (19.5%)	46 (24.5%)	
1	64 (56.6%)	108 (57.4%)	
2	26 (23.0%)	32 (17.0%)	
**OCS user**	30 (26.5%)	81 (43.1%)	0.004
**OCS dose (mg)**	8 (5,10)	10 (7,12)	0.012
**ICS dose (BDP µg equivalent)**	2000 (2000,2000)	2000 (2000,2000)	0.573
**LAMA User**	40 (35.4%)	104 (55.3%)	<0.001
**ACQ-7 score**	0.8 (0.4)	2.7 (0.8)	<0.001
**AQLQ total score**	6.1 (0.7)	4.2 (1.2)	<0.001

* Sputum data was available in 123 subjects at baseline; [†] Ethnicity as per Global Lung Initiative

ACQ = Asthma control questionnaire; BDP = beclometasone dipropionate; BMI = Body mass index; FeNO = fractional exhaled nitric oxide; FEV1 = forced expiratory volume in 1 second; FVC = Forced vital capacity; ICS = inhaled corticosteroid; ITU = intensive treatment unit; LAMA = long-acting muscarinic antagonists; OCS = oral corticosteroid; PEFR = peak expiratory flow rate

Table E7b Demographics, medical history, lung function, biomarkers, corticosteroid treatment and patient reported outcomes in ACQ≥1.5 by treatment arm

	Biomarker	Symptom	P-value
**Number of Patients**	148	40	
**Age At Inclusion (y)**	54.3 (12.6)	56.3 (12.3)	0.376
**Gender**			0.290
Female	102 (68.9%)	31 (77.5%)	
Male	46 (31.1%)	9 (22.5%)	
**Ethnicity** [ƚ]			0.929
Caucasian	135 (91.2%)	38 (95.0%)	
South East Asian	6 (4.1%)	1 (2.5%)	
Other	5 (3.4%)	1 (2.5%)	
Mixed	1 (0.7%)	0 (0.0%)	
African	1 (0.7%)	0 (0.0%)	
**BMI (kg/m^2)**	33.2 (7.5)	33.7 (6.5)	0.698
**Smoking Status**			0.050
Never smoked	115 (77.7%)	25 (62.5%)	
Ex-smoker	33 (22.3%)	15 (37.5%)	
**Working Status**			0.477
Not working due to asthma related ill health	37 (25.0%)	11 (27.5%)	
Not Working due to other cause	38 (25.7%)	14 (35.0%)	
Working part-time due to related ill health	9 (6.1%)	1 (2.5%)	
Working part-time due to other cause	15 (10.1%)	1 (2.5%)	
Student	2 (1.4%)	0 (0.0%)	
Full time	46 (31.1%)	13 (32.5%)	
**Atopic Disease**	99 (66.9%)	28 (70.0%)	0.750
**Hospital Admissions For Asthma In Last Year**	0.0 (0.0,0.0)	0.0 (0.0,0.0)	0.169
**A&E Visits In Last Year**	0.0 (0.0,1.0)	0.0 (0.0,0.0)	0.733
**GP Visits For Asthma In The Last Year**	1.5 (0.0,4.0)	2.0 (0.0,3.0)	0.837
**Rescue Courses Of Oral Steroids In The Last Year**	3.0 (1.0,5.0)	3.0 (1.0,4.0)	0.748
**Prior Admission for Asthma to ITU**	39 (26.4%)	8 (20.0%)	0.410
**Number Of Prior Admissions For Asthma to ITU**	1.0 (1.0,3.0)	1.0 (1.0,2.5)	0.767
**Ever Been Ventilated**	21 (14.2%)	4 (10.0%)	0.786
**Rhinitis**	103 (69.6%)	25 (62.5%)	0.393
**Eczema**	53 (35.8%)	13 (32.5%)	0.697
**Nasal polyps**	33 (22.3%)	11 (27.5%)	0.490
**Prior nasal surgery**	35 (23.6%)	8 (20.0%)	0.626
**Oesophageal reflux**	96 (64.9%)	30 (75.0%)	0.226
**Aspirin sensitivity**	24 (16.2%)	9 (22.5%)	0.354
**Depression / anxiety**	58 (39.2%)	17 (42.5%)	0.704
**Hypertension**	46 (31.1%)	19 (47.5%)	0.053
**Osteoporosis / osteopenia**	45 (30.4%)	8 (20.0%)	0.194
**Osteoarthritis**	43 (29.1%)	10 (25.0%)	0.613
**Hypercholesterolaemia**	27 (18.2%)	9 (22.5%)	0.544
**Diabetes**	21 (14.2%)	3 (7.5%)	0.261
**Cataracts**	16 (10.8%)	3 (7.5%)	0.538
**Obstructive sleep apnoea**	12 (8.1%)	1 (2.5%)	0.215
**Ischaemic heart disease**	5 (3.4%)	2 (5.0%)	0.631
**Peptic ulcer**	5 (3.4%)	0 (0.0%)	0.239
**Stroke**	2 (1.4%)	2 (5.0%)	0.156
**Chronic kidney disease**	3 (2.0%)	2 (5.0%)	0.300
**Glaucoma**	3 (2.0%)	0 (0.0%)	0.364
**Myocardial infarction**	1 (0.7%)	1 (2.5%)	0.318
**FEV_1**	2.0 (0.7)	1.9 (0.7)	0.333
**% Predicted FEV_1**	70.6 (18.3)	71.0 (20.1)	0.902
**FVC**	3.1 (0.8)	3.0 (0.9)	0.476
**% Predicted FVC**	85.6 (15.1)	88.5 (18.9)	0.298
**FEV_1/FVC**	0.65 (0.12)	0.64 (0.13)	0.411
**PEFR (L/m)**	349.8 (109.6)	322.7 (126.6)	0.184
**Sputum eosinophils (%)***	1.0 (0.3,4.5)	2.0 (0.5,4.3)	0.584
**Sputum neutrophils (%)**	67.4 (42.1,76.0)	66.9 (45.0,84.4)	0.480
**Sputum lymphocytes (%)**	0.5 (0.0,1.4)	0.1 (0.0,1.2)	0.243
**Macrophage sputum (%)**	23.2 (12.3,39.8)	25.1 (6.6,41.9)	0.800
**FeNO (ppb)**	20 (13,30)	18 (12,27)	0.245
**Blood eosinophil count (10^9 cells/L)**	0.19 (0.10,0.30)	0.27 (0.13,0.42)	0.038
**Periostin (ng/ml)**	51.6 (14.9)	51.9 (15.4)	0.914
**Composite score**			0.554
0	37 (25.0%)	9 (22.5%)	
1	82 (55.4%)	26 (65.0%)	
2	27 (18.2%)	5 (12.5%)	
**Baseline Medications**			
**OCS User**	64 (43.2%)	17 (42.5%)	0.933
**OCS Dose (mg)**	10 (8,12)	8 (5,10)	0.183
**ICS Dose (BDP µg equivalent)**	2000 (2000,2000)	2000 (2000,2000)	0.815
**LAMA User**	80 (54.1%)	24 (60.0%)	0.502
**Patient Reported Outcomes**			
**ACQ-7 Score**	2.7 (0.8)	2.8 (0.8)	0.478
**AQLQ Total Score**	4.2 (1.1)	3.9 (1.2)	0.117

* Sputum data was available in 73 subjects at baseline; [ƚ] Ethnicity as per Global Lung Initiative

ACQ = Asthma control questionnaire; BDP = beclomethasone dipropionate; BMI = Body mass index; FeNO = fractional exhaled nitric oxide; FEV1 = forced expiratory volume in 1 second; FVC = Forced vital capacity; ICS = inhaled corticosteroid; ITU = intensive treatment unit; LAMA = long-acting muscarinic antagonists; OCS = oral corticosteroid; PEFR = peak expiratory flow rate

5. ADVERSE EVENTS

In relation to adverse events, the terms ‘severe’ and ‘serious’ were not synonymous. The severity, or intensity, of an AE referred to the extent to which an AE affected the patient’s daily activities:

- Mild: An AE usually transient in nature and generally not interfering with normal activities.
- Moderate: An AE that is sufficiently discomforting to interfere with normal activities
- Severe: An AE that is incapacitating and prevents normal activities.

Serious adverse events (SAEs) suspected by the investigator to be associated with study procedures and which are unexpected will need to be reported immediately to the sponsor (i.e. within 24 hours of knowledge of the event) during this observational study.

An SAE was any AE that is assessed by the investigator to fulfil any of the following criteria:

- Results in death (i.e. the AE actually causes or leads to death)
- Is immediately life threatening (i.e., the AE, in the view of the investigator, places the patient at immediate risk of death)
- Requires inpatient hospitalisation or prolongation of existing hospitalisation
- Results in persistent or significant disability/incapacity, (i.e., the AE results in substantial disruption of the patient’s ability to conduct normal life functions)
- Results in a congenital anomaly/birth defect
- Is otherwise considered medically significant by the investigator

There was no difference in Mild/Moderate AEs between study groups (rate ratio [symptom:biomarker] 1.10; 95% CI 0.96 – 1.26). There was no difference in severe AEs between study groups (rate ratio [symptom:biomarker] 1.26; 95% CI 0.78 – 2.05).

There was an increased rate of reported serious AEs in the control group compared to the biomarker strategy group (rate ratio [symptom:biomarker] 1.64; 95% CI 1.04 – 2.60). Reported serious AEs are listed in the table below. There was no difference in adjusted rate for hospitalisations for asthma between the study groups (see Table 4 main text).

Table E8: Table of reported serious adverse events by treatment arm

Biomarker	Control
Asthma exacerbation	Asthma exacerbation
Pneumonia	Atrial fibrillation
Cellulitis	Bronchopneumonia and Sepsis
Chest infection	Cauda Equina syndrome
Pregnancy	Cellulitis
Vulvar cancer	Chest Infection
Abdominal pain	Cholera
Acquired haemophilia	Collapsed during spirometry
Altered urea and electrolytes	Colorectal cancer
Angina	Diarrhoea
Bronchiectasis	EUA and Dolormes procedure
Chest Pain	Gastroenteritis
Costochondritis	Heart Failure
Critical ischaemia of right lower leg	Left bundle branch block
Dog bite	Mental Health
Dupuytren's contracture	Myocarditis
Epigastric Discomfort	PoTS syndrome
Gallbladder removal	Pneumonia
Influenza	Renal impairment
Kidney stones	Sinus surgery
Knee arthroscopy	Viral Chest Infection
Knee replacement	Vomiting and loose stool
Psychiatric event	
Shoulder dislocation	
Suspected Sepsis	
Synovitis	
Tight chest after sputum induction	
Tooth Abscess	
Urinary Tract Infection	
Unstable CLL	
Worsening osteoarthritis	

6. REFERENCES

1. Hanratty CE, Matthews JG, Arron JR, Choy DF, Pavord ID, Bradding P, Brightling CE, Chaudhuri R, Cowan DC, Djukanovic R, Gallagher N, Fowler SJ, Hardman TC, Harrison T, Holweg CT, Howarth PH, Lordan J, Mansur AH, Menzies-Gow A, Mosesova S, Niven RM, Robinson DS, Shaw DE, Walker S, Woodcock A, Heaney LG; RASP-UK (Refractory Asthma Stratification Programme) Consortium. A randomised pragmatic trial of corticosteroid optimization in severe asthma using a composite biomarker algorithm to adjust corticosteroid dose versus standard care: study protocol for a randomised trial. Trials. 2018 Jan 4;19(1):5.
2. Hanania et al. Omalizumab in severe allergic asthma inadequately controlled with standard therapy: a randomized trial. Ann Intern Med 2011 154:573
3. Corren J et al. Lebrikizumab treatment in adults with asthma. N Engl J Med. 2011;365(12):1088-98
4. Nicola A Hanania, Michael Noonan, Jonathan Corren, Phillip Korenblat, Yanan Zheng, Saloumeh K Fischer, Melissa Cheu, Wendy S Putnam, Elaine Murray, Heleen Scheerens, Cecile TJ Holweg, Romeo Maciuca, Sarah Gray, Ramona Doyle, Dana McClintock, Julie Olsson, John G Matthews, Karl Yen. Lebrikizumab in moderate-to-severe asthma: pooled data from two randomised placebo-controlled studies. Thorax 2015 Aug;70(8):748-56. doi: 10.1136/thoraxjnl-2014-206719.
